# Combined effects of Lenvatinib and zinc oxide nanoparticles by promoting apoptosis and anti-proliferative activity in mice bearing Ehrlich solid tumors

**DOI:** 10.1186/s11671-026-04802-4

**Published:** 2026-07-20

**Authors:** Faten Zahran, Shimaa El-Metwaly, Ahmed A.Shaaban, Ahmed Nabil

**Affiliations:** 1 Biochemistry Department, Faculty of Science, Zagazig University, Zagazig, Egypt; 2 Pharmacology and Toxicology Department, Faculty of Pharmacy, Mansoura University, Mansoura, Egypt; 3 Biotechnology and Life Sciences Department, Faculty of Postgraduate Studies for Advanced Sciences (PSAS), Beni-Suef University, Beni-Suef, Egypt

**Keywords:** Ehrlich, Lenvatinib, ZnO-NPs, Caspase-3, Ki-67

## Abstract

**Objective:**

The objective of this study is to evaluate the anticancer activity of lenvatinib and zinc oxide nanoparticles (ZnO-NPs) separately and in combination and whether ZnO-NPs can reduce resistance to lenvatinib treatment.

**Methods:**

TEM, SEM, Zeta, DLS, UV, FTIR, and XRD performed to characterize ZnO-NPs. Fifty mature female Swiss albino mice bearing solid Ehrlich carcinoma (SEC) were randomly divided into five equal groups: Negative control, SEC, Lenvatinib, ZnO-NPs, and Lenvatinib + ZnO-NPs. All treatments were administered for 4 weeks.

**Results:**

A decrease in tumor volume (458.6 mm^3^**)** and an increase in tumor growth inhibition (67.6%) were observed in the combination groups. In the combination group, there was a decrease in serum ALT and AST levels. Besides, there was a significant increase in reactive oxygen species (ROS), malondialdehyde (MDA) and tumor necrosis factor-alpha (TNF-α) accompanied by depletion in superoxide dismutase (SOD) and catalase, in tumor tissues. Notably, the anti-apoptotic marker Bcl-2 significantly decreased, while caspase-3 and caspase-9 levels in blood samples and caspase-3 expression in tumor tissues increased significantly. Cell cycle research revealed a G2/M phase arrest in tumor samples. Additionally, Cyclin D1 expression (0.63) was significantly reduced by western blotting, and immunohistochemical staining showed lower Ki-67 levels (46.6%). Elevation of apoptosis and necrosis was confirmed by histopathological analysis of tumor sections. Also, histopathological examination of major organs (liver, kidney, heart, and spleen) showed no discernible damage.

**Conclusion:**

According to these results, zinc oxide nanoparticles may increase lenvatinib's anticancer effectiveness by promoting apoptosis and inhibiting proliferation. Future clinical research may employ the combination because it seems to be both safe and effective.

**Supplementary Information:**

The online version contains supplementary material available at 10.1186/s11671-026-04802-4.

## Introduction

Cancer remains one of the most significant global public health challenges, accounting for substantial morbidity and mortality worldwide. Every day, more than 52,900 individuals are diagnosed with cancer, while over 27,000 deaths are attributed to the disease [1]. Cancer development is driven by the accumulation of genetic and epigenetic alterations that confer hallmark characteristics to malignant cells, including uncontrolled proliferation, resistance to apoptosis, immune evasion, replicative immortality, invasiveness, and metastatic potential. In addition to tumor cell intrinsic factors, interactions between cancer cells and the surrounding tumor microenvironment (TME) play a critical role in tumor initiation, progression, and metastasis. These interactions facilitate the acquisition of malignant phenotypes and establish a supportive environment for tumor growth and survival [2]. Rapidly proliferating tumors require a continuous supply of oxygen and nutrients, as well as efficient removal of metabolic waste products. These demands are largely met through the formation of new blood vessels, a process known as angiogenesis, which is considered a hallmark of cancer progression and a key determinant of tumor growth and dissemination [3]. Consequently, targeting tumor angiogenesis has emerged as an important therapeutic strategy in cancer treatment.

Recent advances in cancer therapy have substantially improved treatment efficacy and precision. Nevertheless, major limitations continue to compromise clinical outcomes. Surgical intervention is highly effective for localized tumors; however, its success is often restricted by tumor size, anatomical location, and the presence of metastases [4]. Similarly, chemotherapy and hormone therapy frequently encounter treatment resistance resulting from genetic mutations, alterations in signaling pathways, and tumor heterogeneity, thereby reducing their long term effectiveness [5]. Furthermore, radiation therapy can cause significant damage to healthy tissues and organs surrounding the tumor site [6]. These limitations underscore the need for more effective and targeted therapeutic approaches.

Tyrosine kinase inhibitors (TKIs) have emerged as a major class of targeted anticancer agents and have transformed the management of several malignancies. These agents exert their therapeutic effects by binding to the ATP binding site of tyrosine kinases, thereby preventing substrate phosphorylation and inhibiting downstream signaling pathways involved in cellular proliferation, survival, and angiogenesis. Through suppression of these signaling cascades, TKIs effectively inhibit tumor growth and metastatic progression [7].

Among these agents, lenvatinib, an orally active multitarget receptor tyrosine kinase inhibitor, has received approval from the United States Food and Drug Administration for the treatment of several cancers, including differentiated thyroid cancer, advanced renal cell carcinoma, and hepatocellular carcinoma [8]. Lenvatinib exerts its antitumor activity through inhibition of multiple signaling pathways, including vascular endothelial growth factor receptors (VEGFR1–3), fibroblast growth factor receptors (FGFR1–4), platelet derived growth factor receptor-α (PDGFR-α), the rearranged during transfection (RET) protooncogene, and the stem cell factor receptor (KIT) [9]. Through simultaneous blockade of these targets, lenvatinib effectively suppresses angiogenesis and tumor growth. However, despite its initial clinical efficacy, the majority of patients eventually develop acquired resistance within approximately two years of treatment, limiting long-term therapeutic benefits [10]. Moreover, treatment-associated adverse effects, including hypertension, diarrhea, fatigue, asthenia, anorexia, and weight loss, frequently necessitate dose reduction or treatment discontinuation [11]. Therefore, the development of combination strategies capable of enhancing lenvatinib efficacy, minimizing toxicity, and overcoming therapeutic resistance remains an important clinical objective.

Nanotechnology has emerged as a promising platform for improving cancer treatment by enhancing drug bioavailability, enabling targeted drug delivery, and reducing systemic toxicity [12]. Nanoparticle based systems have revolutionized anticancer drug development owing to their favorable pharmacokinetic properties, prolonged circulation time, increased tumor accumulation, and improved therapeutic index. Following systemic administration, nanoparticles preferentially accumulate within tumor tissues through enhanced permeability and retention (EPR) effects, facilitated by abnormal tumor vasculature and impaired lymphatic drainage [13].

Among various nanomaterials, zinc oxide nanoparticles (ZnO-NPs) have attracted considerable attention because of their unique physicochemical properties, including biocompatibility, large surface area, small particle size, and ease of surface modification. These characteristics make ZnO-NPs attractive candidates for cancer therapy, as they can enhance localized drug delivery, improve therapeutic efficacy, and modulate the tumor microenvironment. In addition, ZnO-NPs exhibit remarkable stability under physiological conditions and can be engineered to achieve controlled and stimuli-responsive drug release [14]. Beyond their applications in targeted drug delivery, ZnO-NPs have demonstrated broad biological activities, including antimicrobial, anticancer, and vaccine-related applications [15]. Importantly, ZnO-NPs possess intrinsic anticancer activity. Previous studies have shown that ZnO-NPs induce apoptosis in breast cancer cells through activation of the mitochondrial apoptotic pathway [16]. Their cytotoxic effects are primarily mediated through excessive generation of reactive oxygen species (ROS), leading to oxidative stress, mitochondrial dysfunction, and subsequent cell death. When ROS production exceeds the antioxidant defense capacity of cancer cells, severe cellular damage occurs, ultimately resulting in apoptosis or necrosis [17]. These mechanisms differ from those of anti-angiogenic agents such as lenvatinib, suggesting the possibility of complementary or synergistic therapeutic effects when both agents are used in combination.

Supporting this concept, nanoparticle-based delivery systems have been reported to enhance the antitumor activity of TKIs. For example, Jayalakshmi et al. demonstrated that gum Arabic stabilized zein nanoparticles loaded with erlotinib or gefitinib significantly improved anticancer efficacy by increasing ROS generation and inducing mitochondrial membrane depolarization in treated cancer cells [18]. Therefore, combining lenvatinib with bioactive nanoparticles such as ZnO-NPs may provide a dual therapeutic approach that simultaneously suppresses angiogenesis and promotes apoptosis-mediated tumor cell death.

Solid Ehrlich carcinoma (SEC) is a highly aggressive, rapidly proliferating, and transplantable murine tumor model that closely resembles several characteristics of human malignancies. Owing to its reproducible growth pattern, high malignancy, and lack of spontaneous regression, SEC is widely employed in preclinical studies investigating novel anticancer therapies [19].

Despite the growing interest in nanoparticle-based cancer therapies, studies evaluating the combined therapeutic potential of ZnO-NPs and anti-angiogenic agents such as lenvatinib remain limited, particularly in solid Ehrlich carcinoma models. Therefore, the present study aimed to investigate the antitumor efficacy of lenvatinib and ZnO-NPs, administered individually or in combination, in SEC bearing mice. In addition, the potential toxicological effects of these treatments on major organs, including the liver, kidney, heart, and spleen, were assessed through histopathological examination using hematoxylin and eosin (H&E) staining.

## Materials and methods

### Drugs and chemicals

Lenvatinib (C_21_H_19_ClN_4_O_4_, Mw = 426.9 g/mol, CAS No. 417716–92-8) was purchased from Eisai Co., Ltd., Japan. Potassium hydroxide (KOH, Mw = 56.105 g/mol, CAS No. 1310–58-3) was provided by Sigma-Aldrich Chemical Co. (St. Louis, MO, USA). Zinc nitrate hexahydrate (Zn(NO_3_)_2_.6H2O, Mw = 297.49 g/mol, CAS No. 10196–18-6) was also provided by Sigma-Aldrich Chemical Co. (St. Louis, MO, USA).

### Lenvatinib preparation

Lenvatinib was uniformly suspended in 0.5% carboxymethylcellulose/saline solution.

### Synthesis of the ZnO-NPs

The direct precipitation approach was used to create ZnO-NPs using potassium hydroxide and zinc nitrate as precursors. This study utilized deionized water to produce aqueous solutions of (Zn(NO_3_)_2_.6H2O, 0.2 M) and (KOH, 0.4 M). The KOH solution was cautiously added to the room-temperature Zn(NO_3_)_2_.6H2O solution while vigorously stirring to create a white suspension. Following 20 min of centrifugation at 5000 rpm at room temperature (25 ^◦^C), this white suspension was rinsed three times with distilled water and subsequently sterilized with pure alcohol. To create the white solid ZnO-NPs, the product was broken up into little pieces, dried for an hour at 220 ^◦^C, and then left to sit at 500 ^◦^C for four hours [20].

### Characterization of the ZnO-NPs

ZnO-NPs were fully characterized by Transmission Electron Microscopy (TEM), Scanning Electron Microscopy (SEM), Zeta potential analysis (Zeta), dynamic light scattering (DLS), ultraviolet (UV), Fourier Transform Infrared Spectroscopy (FTIR), and X-Ray Diffraction (XRD). A transmission electron microscope (A JEOL JEM-2100, Japan) and a scanning electron microscope (JEOL JSM-6490LV, Japan) were used to examine and determine size and morphology. The Zetasizer Ultra Red Label (Malvern Instruments, UK) was utilized as well to evaluate the surface zeta potential. The hydrodynamic particle size and polydispersity index (PDI) of the produced ZnO nanoparticles were assessed at 25 ± 2 °C via DLS utilizing a Zetasizer Advance Series Ultra Red Label instrument (Malvern Panalytical, UK). A UV–Visible Spectrophotometer (Shimadzu, 9000i, Japan) was used to examine the optical characteristics of the produced ZnO- NPs. UV and visible (VIS) portions of the spectrum can be used to assess substances based on how much light they absorb. Usually, absorbance or percent transmittance (%T) are used to display the data. To identify the absorption edge (λ) and light-absorption features, absorbance spectra were acquired between 200 and 800 nm. FTIR spectral analysis was performed using a Bruker FTIR spectrometer (Invenio S, Germany). The spectra were recorded over the wavenumber range of 350–4000 cm⁻^1^ with a spectral resolution of 4 cm⁻^1^ and 64 accumulated scans for each measurement [21]. A Bruker D8-Advance device operating at 40 kV and 50 mA for CuKα (λ = 1.54056 ˚A) radiation, with a scanning speed of 50°/min and a step width of 0.01° in 2θ in the range of 5° to 80°, was used to obtain powder XRD patterns. The crystalline structure and phase composition of the material are revealed by XRD examination.

### Solid tumor induction in mice

Ehrlich Ascites Carcinoma (EAC) cells were acquired from Cairo University's National Cancer Institute (Giza, Egypt). For solid tumor induction, two million EAC cells were implanted subcutaneously into the right thigh of the mice's hind limb. Before being injected into mice, the trypan blue assay was used to measure the cell viability, and a hemocytometer was used to count the cells. Within 12 days of implantation, a substantial tumor mass formed [22].

### Experimental design

The Institutional Animal Care and Use Committee of Zagazig University accepted the study design (permission number ZU-IACUC/1/F/94/2023), and this work adhered to the ethical standards for laboratory animal research. Under regular laboratory circumstances, female Swiss Albino mice weighing between 22 and 30 g were let to acclimatize for one week with unrestricted access to food and water. Fifty female Swiss mice were randomly separated into five group, and each group contains ten mice. Group 1 was regarded as negative control. Group 2 to group 5 were administered 2 × 10^6^ EAC subcutaneously. Group 2 was regarded as positive control. Group 3 received 10 mg/kg/day lenvatinib by oral gavage according to reference [23], and group 4 was administered 5 mg/kg of ZnO-NPs I.P/day according to reference [24]. Group 5 administered 10 mg/kg/day lenvatinib plus 5 mg/kg/day of ZnO-NPs. The doses of ZnO-NPS and Lenvatinib were selected according to previous studies. All groups began their treatment protocol on day 12 and continued until day 28 after implantation.

### Tumor volume (V) and percentage tumor growth inhibition (% TGI)

The tumor volume was recorded using Calipers by the same blinded observer twice a week. The volume of the developing tumor mass was determined utilizing the subsequent equation:

$$ {\text{tumor volume mm}}^{3} = 0.5 \, \times {\text{ length }} \times {\text{ width}}^{2} $$

The percentage of tumor growth inhibition (% TGI) estimated served as an indication for drug efficacy.

$$ \% {\text{ TGI}} = { 1}00 - \left( {{\mathrm{T}}/{\text{C }} \times {1}00} \right) $$

T indicates the mean relative tumor volume (RTV) of the treated groups, while C stands for the mean RTV of the control group. RTV is obtained as Vx/V1, where V1 is the tumor volume at the start of the therapy and Vx is the tumor volume at each stage of the experiment prior to mice scarification [25].

### Collection and preparation of samples

Considering pain management, the initial sedation was achieved using halothane, followed by intraperitoneal administration of ketamine (75 mg/kg) and xylazine (10 mg/kg). All animals were euthanized via decapitation after anesthetization, and their blood was divided into two parts. The first sample was placed in an EDTA tube for hematological and flowcytometry examination. The second half was collected in plain tubes and centrifuged to separate the serum. The tumor mass was carefully excised and separated into three sections. The first was stored in liquid nitrogen, while the second was kept at -80°C. The third specimen was submerged in 10% formaldehyde for histological examination.

### Hematological and biochemical analysis

A common automated counter utilized to determine red blood cells, hemoglobin (Hb), white blood cells, and platelet count was the Mindray BC-2800 Vet analyzer (Shenzhen Mindray Bio-Medical Electronics Co., Shenzhen, China). Blood urea, serum creatinine, aspartate aminotransferase (AST), and alanine amino transferase (ALT) levels were estimated using an automatic clinical chemistry analyzer (BS-600M, Mindray, China) [26].

### Assessment of oxidative stress and inflammatory biomarkers in tumor tissue

The levels of catalase, superoxide dismutase (SOD), malondialdehyde (MDA), reactive oxygen species (ROS) and tumor necrosis factor-alpha (TNF-α) were determined in tumor tissue homogenates according to the manufacturers’ protocols by mouse enzyme-linked immunosorbent assay kits: CAT kit (SunLong, Cat. No. SL0747Mo); SOD kit (SunLong, Cat. No. SL0513Mo); MDA kit (Nova Lifetech, Cat. No. CELI-66086m); ROS kit (Nova Lifetech, Cat. No. ELA-E1924m) and TNF-α kit (CUSABIO, Cat. No. CSB-E04741m). Absorbance was read at 450 nm and concentrations determined using standard curves.

### Determination of apoptosis markers caspase-3/9 in blood by flowcytometry

Isolation of peripheral blood mononuclear cells (PBMCs) was performed utilizing Ficoll-Hypaque and then centrifuged for 20 min at 2500 rpm. The cell pellet was rinsed and resuspended in phosphate-buffered saline/bovine serum albumin (PBS/BSA) buffer (pH 7.4, Sigma-Aldrich, Cat. No. L1825; 1% BSA, Thermo Fisher, Cat. No. B14) at a concentration of 1 × 10⁶ cells/mL. An anti-active caspase-3 antibody (BD Biosciences, Cat. No. 550557) or an anti-caspase-9 antibody (Cell Signaling Technology, Cat. No. 508) was added to 100 μl of cell suspension, which was left in the dark for 30 min at room temperature. The cells were centrifuged for five minutes at 2000 rpm after being washed with two milliliters of PBS/BSA, and the supernatant was eliminated. The cell pellet was fixed in 200 μl of 4% paraformaldehyde in PBS and then evaluated utilizing the BD Accuri™ C6 flow cytometer (BD Biosciences, USA). A sequential gating method was used. Debris was eliminated utilizing FSC/SSC criteria. Caspase-3- and caspase-9-positive groups were delineated by histogram analysis, with gates established based on the unstained control. Cells above the fluorescence threshold were deemed caspase-positive (Fig. S2).

### Determination of caspase-3 and cell cycle analysis in tissue by flowcytometry:

Tumor samples from each group were washed using isotonic Tris–EDTA buffer. Following homogenization, samples were centrifuged for 10 min at 1800 rpm. To determine caspase-3 activity, a cell suspension (1 × 10⁶ cells/mL) was rinsed with PBS/BSA, centrifuged for 5 min at 2000 rpm, and suspended again in 100 µL of PBS/Saponin. A 7 µL anti-active caspase-3 antibody (BD Biosciences, Cat. No. 550557) was added, and the samples were subsequently permitted to sit at room temperature for thirty minutes in the absence of light. After washing and centrifugation, cells were fixed in 200 µL of 4% paraformaldehyde in PBS. The analysis was conducted utilizing a BD Accuri™ C6 flow cytometer (BD, USA) [27]. To assess the cell cycle, cells were immersed in 70% ethanol on ice for ≥ 2 h, rinsed with PBS, and dyed with PBS including 100 µg/mL RNase A, 50 µg/mL propidium iodide, and 0.1% Triton X-100 [28]. A progressive gating method was employed for cell cycle analysis. Debris was initially eliminated utilizing FSC-A/SSC-A standards. Cell cycle was assessed utilizing a PI fluorescence histogram. Cell populations were further quantified into Sub-G1, G0/G1, S, and G2/M phases based on DNA content distribution (Fig. S3).

### Detection of BCL2 and cyclin D1 by western blotting

The ReadyPrep™ Protein Extraction Kit (Bio-Rad, Cat. No. 163–2086) was utilized to obtain total proteins from tumor tissue following the manufacturer's guidelines. Using the Bradford Protein Assay Kit (Bio Basic Inc., Cat. No. SK3041), protein concentrations were determined. TGX Stain-Free™ FastCast™ Acrylamide Gels (Bio-Rad, Cat. No. 161–0181) were used to separate the proteins that were obtained, and BioRad Trans-Blot Turbo was used to transfer them to a nitrocellulose membrane. To block the membrane, it was incubated in tris-buffered saline with 3% BSA and Tween 20 (TBST) for an hour at room temperature. Primary antibodies against Bcl-2 (mouse monoclonal, clone C-2, sc-7382) and Cyclin D1 (mouse monoclonal, clone A-12, sc-8396), both from Santa Cruz Biotechnology (Dallas, TX, USA), were incubated with membrane at 4 °C overnight. Next, horseradish peroxidase)HR (conjugated goat anti-rabbit secondary antibody was applied to the membrane (Novus Biologicals) at room temperature for an hour. Protein bands were seen with Clarity™ Western ECL substrate (Bio-Rad, Cat. No. 170–5060), and chemiluminescent signals were captured with ChemiDoc MP imaging equipment. The band intensities of Bcl-2 and Cyclin D1 were quantified using densitometric analysis with ImageJ software and normalized to the corresponding β-actin band intensity of the same sample. The relative expression of protein is given as the target protein/β-actin ratio. β-actin was utilized as loading control to confirm the same amount of protein loaded in each lane. [29].

### Histopathological analysis

Fresh liver, kidney, heart, spleen, and solid Ehrlich tumor samples from each experimental group were directly preserved in 10% buffered formalin for pathological analysis. Subsequently, the samples were embedded in paraffin, sliced into 5 µm thick sections utilizing a microtome, and stained with hematoxylin and eosin. A blinded pathologist evaluated the stained sections observed through a light microscope (Leica Microsystems, Germany) to look for any histological abnormalities [30]. For tumor tissues, semiquantitative scoring of numbers of tumor giant cells and apoptotic cells were counted besides assessment of mitotic activity, and the degree of tumor necrosis in six sections from each animal in the different groups by the same blinded pathologist.

### Immunohistochemical staining of Ki-67

Sections of 4 μm thick formalin-fixed, paraffin-embedded (FFPE) tissue were deparaffinized in xylene and then rehydrated with graded ethanol. A pressure cooker and Heat-induced Epitope Retrieval (HIER) with Trilogy™ buffer (Cell Marque, Cat. No. 920P-06) were used to extract the antigens. Sections were treated for 60 min with rabbit monoclonal anti-Ki-67 antibody (clone MD288R, Medaysis, Cat. No. RM0255RTU7) after endogenous peroxidase was blocked with 3% hydrogen peroxide. The UltraVision One HRP Polymer system was employed for detection, and a DAB chromogen/substrate mixture was utilized for visualization. Hematoxylin counterstaining, dehydration, and permanent medium mounting were performed on the slides. Lastly, a masked examination of the slides was conducted [31].

### Data analysis

Data analysis was conducted utilizing the Statistical Package for the Social Sciences (SPSS) software (version 19; SPSS, Chicago, Illinois, USA). Data were expressed as mean ± SD. Normality and homogeneity of variance were assessed using the Shapiro–Wilk and Levene tests, respectively. When assumptions were met, comparisons among groups were performed using one-way ANOVA followed by Tukey’s post-hoc test. Statistical significance was set at *P* < 0.05. Each experimental group contained n = 6 animals [32].

## Results

### ZnO-NPs characterization

**Fig. 1:**
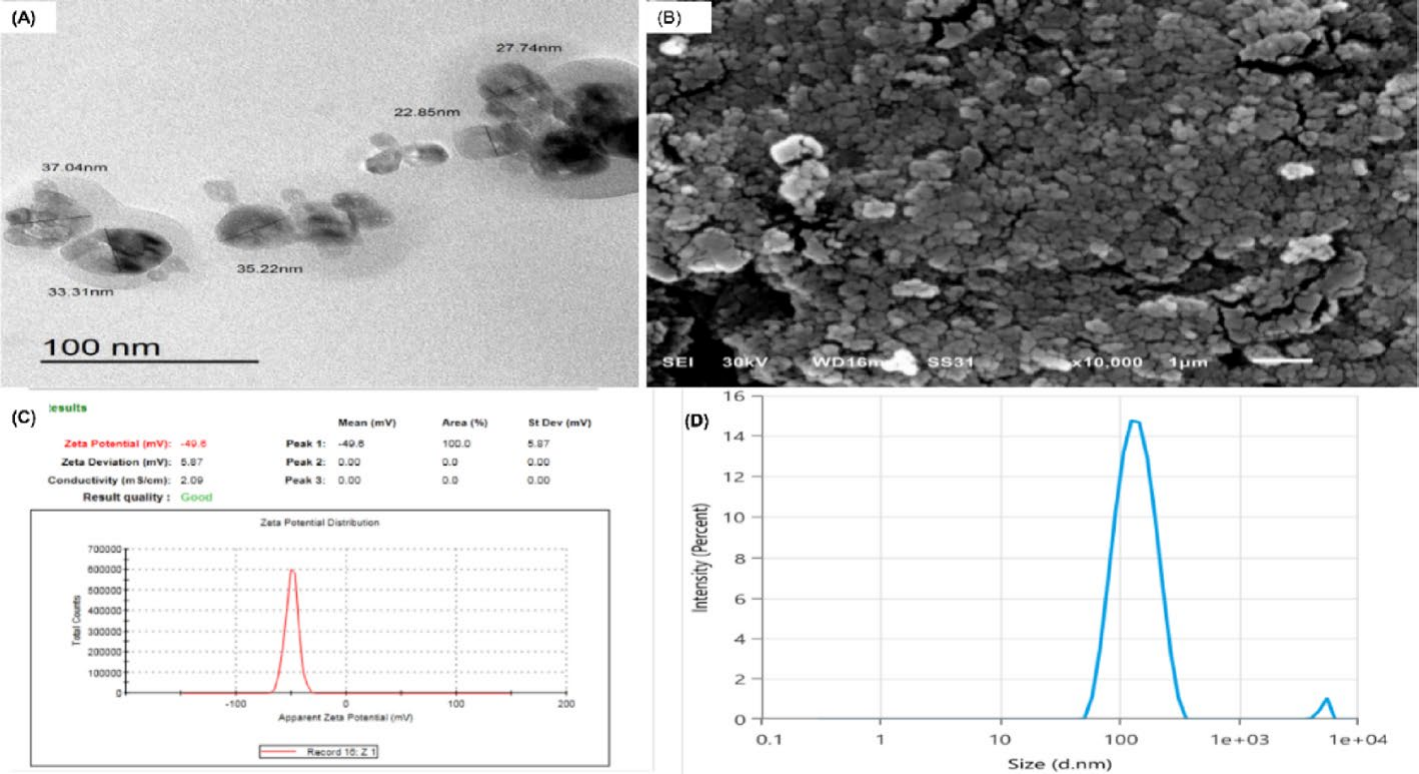
Characterization of ZnO-NPs: (A) TEM image indicating an average particle size of 31.2 nm. Scale bar = 100 nm; (B) SEM image showing surface morphology of manufactured ZnO-NPs. Scale bar = 1 µm; (C) Zeta potential analysis of ZnO-NPs showing -49.6 mV; (D) DLS analysis of ZnO-NPs indicates particle size average = 135 nm, and PDI 0.1

Physicochemical characteristics of ZnO-NPs were studied using various approaches. The particle size and surface charge of ZnO-NPs were confirmed using a zeta potential analyzer, TEM, and SEM. ZnO-NPs exhibited a spheroidal morphology, with an average particle size of 31.2 nm, as indicated by TEM micrographs Fig. 1A. According to Fig. 1C, their conductivity was 2.09 ms/cm, their zeta potential was -49.6 mV exhibited strong stability and low tendency to aggregation, and their zeta deviation was 5.87 mV. After undergoing SEM evaluation, the synthetic ZnO-NPs Fig. 1B showed a uniform grain appearance and a distinct shape devoid of impurities. The average diameter of ZnO-NPs evaluated in distilled water was approximately 135 nm, with a polydispersity index (PDI) of 0.1 Fig. 1D.

**Fig. 2:**
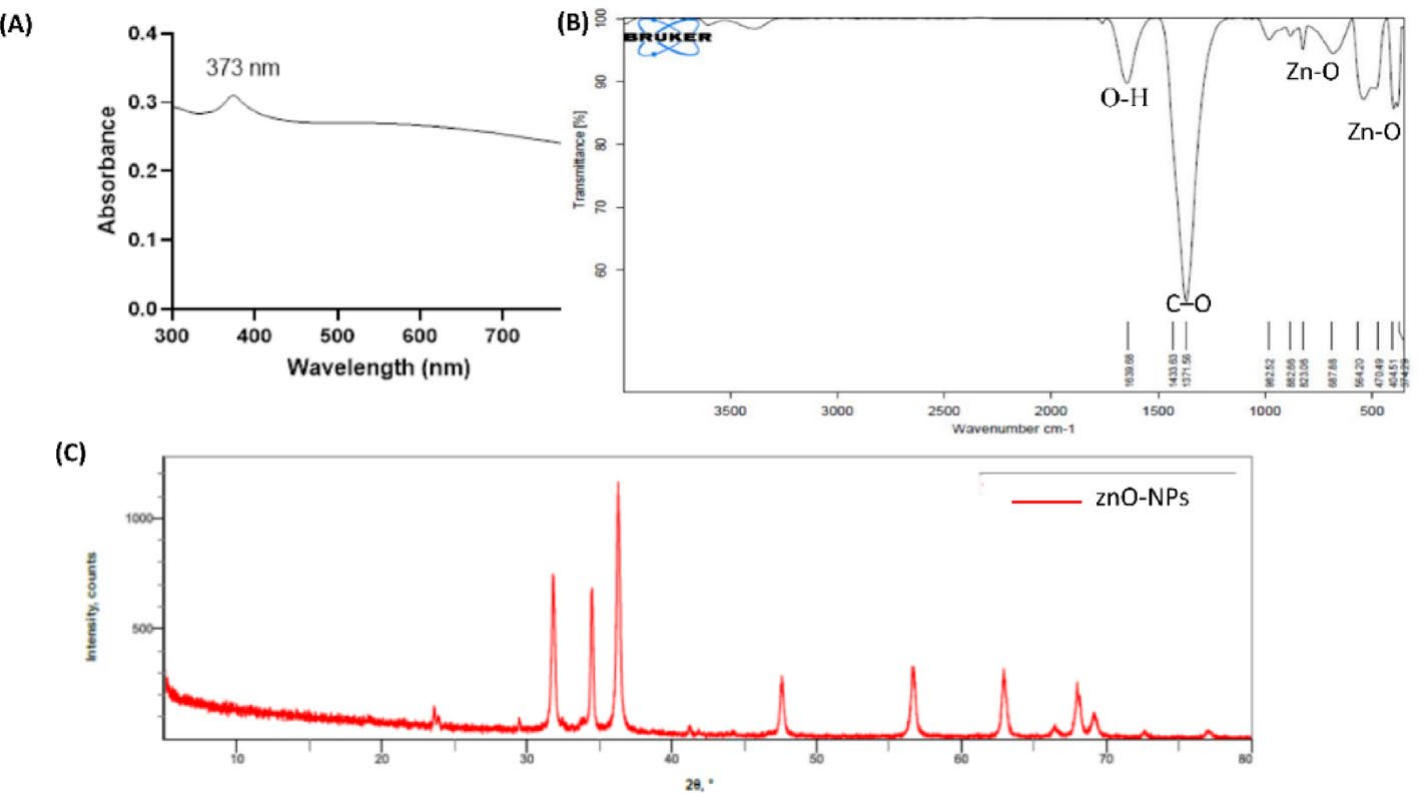
Characterization of ZnO-NPs: (A)UV–vis absorption spectrum of the prepared ZnO-NPs;(B) FTIR spectrum of the prepared ZnO-NPs; (C) XRD pattern of the prepared ZnO-NPs

Figure 2A displays the ZnO nanoparticles' absorption spectra. At around 373 nm, it shows a prominent absorption band. The monodispersed nature of the nanoparticle distribution is also clearly indicated by the notable acute absorption of ZnO-NPs. The principal bands from the produced ZnO nanoparticles' FTIR spectrum are displayed in Table S1 and Fig. 2B along with their corresponding functional group assignments. The presence of adsorbed water on the ZnO surface is shown by the absorption band at 1639.68 cm-1, which is attributed to the bending mode of adsorbed O–H vibrations. The prominent peak in the FTIR spectrum at 1371.56 cm⁻^1^ is attributed to either minor carbonate formation in the alkaline precipitation medium or trace residual carbonate species (C–O antisymmetric stretching of CO_3_^−2^) originating from atmospheric CO2 adsorption during the washing/drying steps [33, 34]. The final calcined product may not have this band, indicating its high purity. Zn–O stretching vibrations are responsible for the distinctive absorption at 823.06 cm⁻^1^, which reflects longitudinal optical phonon modes that signify crystalline order. The strong absorption bands found in the low-frequency range at 564.20 and 470.49 cm-1 are the most intriguing; they are attributed to Zn–O stretching modes and validate the formation of the zinc oxide structure lattice. These bands also represent transverse optical phonon contributions, which further validate the stability of the ZnO lattice.

Figure 2C represents the X-ray diffraction pattern of ZnO- NPs. The diffraction pattern indexed to the hexagonal ZnO phase (Wurtzite Structure) [35, 36] by comparison with the data from the Joint Committee on Powder Diffraction Standards (JCPDS card number 36–1451 and 01–075-0576 [37, 38]) were located at 31.784◦, 34.424◦, 36.284◦,47.560◦, 56.600◦, 62.880◦, 66.3◦, 67.963◦, and 69.091◦ corresponding to the (100), (002), (101), (102), (110), (103), (200), (112), (201) planes, respectively. However, because of their extremely small size, the nanoparticles' XRD patterns are significantly widened. The product's good crystallinity was demonstrated by the strong and narrow diffraction peaks. Additionally, as the produced ZnO nanoparticles only had ZnO peaks as distinctive XRD peaks, they were devoid of contaminants. The remarkable crystallinity of the ZnO nanoparticles is highlighted by the abrupt, powerful peak at 36.33° (101) [39, 40].

### Tumor volume (V), percentage tumor growth inhibition (% TGI)

**Fig. 3:**
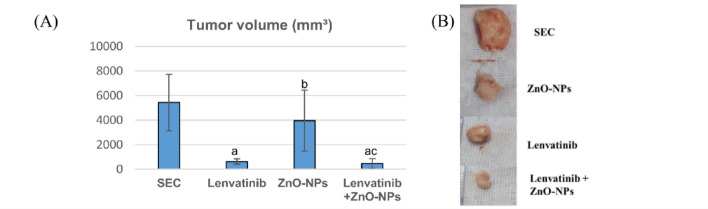
(A) Effect of treatment with Lenvatinib, ZnO-NPs and combinaPtion between Lenvatinib and ZnO-NPs on tumor volume. (B) Excised tumors of all groups after sacrifice. Data are presented as mean ± SD (n = 6). Statistical significance was determined where *P* < 0.05. ^a^ Significant vs SEC, ^b^ Significant vs Lenvatinib, ^c^ Significant vs ZnO-NPs

The volume of the tumor was evaluated to assess the therapeutic efficacy of the various treatments compared to SEC group. As shown in Table 1, there was a significant (*P* = 0.001) decrease in the lenvatinib group and combination group (ZnO-NPs and Lenvatinib) compared to SEC group. On the contrary, ZnO-NPs treated group did not show a significant (*P* = 0.82) decrease in tumor volume in comparison with untreated group Fig. 3A. Additionally, we calculated tumor growth inhibition percentage in all treated groups. We observed significant (*P* = 0.001) increases in lenvatinib group and ZnO-NPs + lenvatinib treated group. In contrast, ZnO-NPs exhibited no significant increase in comparison to SEC group Fig. 3B.

**Table 1:** Effect of lenvatinib, ZnO-NPs and cotherapy of ZnO-NPs + lenvatinib on tumor volume (mm^3^) and tumor growth inhibition

Groups	SEC	Lenvatinib	ZnO-NPs	ZnO-NPs + Lenvatinib
Tumor volume (mm^3^)	5424.1 ± 2301.4	622.6^**a**^ ± 222	3960.8^**b**^ ± 2483.1	458.6^**ac**^ ± 400.2
TGI%	0	76.1^**a**^ ± 23.9	7.1^**b**^ ± 46.2	67.6^**ac**^ ± 26.0

Data are expressed as mean ± SD (n = 6)

^a^ Significant vs SEC, ^b^ Significant vs Lenvatinib, ^c^Significant vs ZnO-NPs, Statistical significance was determined where *P* < 0.05

### Hematological and biochemical parameters

Hematological parameters of normal, tumor, and treated groups are shown in Table 2. There was no significant difference in all parameters (WBCs, RBCs, and platelets) except hemoglobin. In solid Ehrlich tumor group, there was a notable (*P* = 0.001) decrease in hemoglobin in comparison to normal group, but after cotreatment (ZnO-NPs + Lenvatinib), there was a substantial increase compared to solid Ehrlich carcinoma bearing mice. In addition, some changes induced by tumor in liver and kidney biomarkers, such as AST and ALT, urea, and creatinine, strongly suggested liver and kidney damage in the untreated SEC group relative to the normal group. However, there was a notable improvement in liver enzymes ALT and AST (*P* = 0.001) in all treated groups especially in combination treated group (ZnO-NPs + Lenvatinib). In kidney functions, a notable reduction in creatinine levels was observed in all treatment groups, but regarding urea there was a notable restoration in Lenvatinib and cotreatment group (ZnO-NPs + Lenvatinib).

**Table 2:** Effect of lenvatinib, ZnO-NPs and cotherapy of ZnO-NPs + Lenvatinib on hematological and biochemical parameters

Parameters	Negative control	SEC	Lenvatinib	ZnO-NPs	ZnO-NPs + Lenvatinib
Leucocytes (cells × 103/µL)	2.6 ± 0.6	12.5 ± 3.9	21.3^a^ ± 14.4	12.03 ± 3.28	6.2 ± 1.37
RBCs (cells × 106/ µL)	8.2 ± 0.3	5.9^a^ ± 0.8	6.6 ± 0.8	6.04^a^ ± 0.88	6.86 ± 1.29
HB (g/dL)	13.6 ± 1.07	9.1^a^ ± 0.2	9.5^a^ ± 0.4	9.8^a^ ± 0.92	11.5^ab^ ± 0.74
PLT (× 103/µL)	591.8 ± 345.8	525 ± 309	786.5 ± 122.3	1065.3 ± 552.3	1132.8 ± 276.93
ALT (U/L)	43.7 ± 11.8	314^a^ ± 47	49.0^b^ ± 21.6	41.7^b^ ± 35	24^b^ ± 10.9
AST (U/L)	193.6 ± 79	443.7^a^ ± 106	109.0^b^ ± 26.3	181.9^b^ ± 56.4	95.8^b^ ± 47.2
Creatinine (mg/dl)	0.56 ± 0.1	0.96^a^ ± 0.26	0.53^b^ ± 0.11	0.43^b^ ± 0.051	0.45^b^ ± 0.13
Urea (mg/dl)	34.0 ± 8.1	59.3^a^ ± 6.5	37.3^b^ ± 7.2	42.2 ± 7.79	35.1^b^ ± 6.3

Data is represented as mean ± SD (n = 6). ^a^ significant against negative control at (*P* < 0.05), ^b^ significant against SEC group at (*P* < 0.05**)**

### Assessment of oxidative stress and inflammatory biomarkers in tumor tissue

Our results exhibited significant elevation in ROS and MDA levels, as well as a marked depletion in antioxidant enzymes SOD and catalase in all treated groups in comparison to mice bearing solid tumor. In addition, there was a significant increase in TNF-α levels in all treated group in compared to SEC. cotreatment group of ZnO-NPs + Lenvatinib showed higher elevation in ROS, MDA and TNF-α, accompanied by the greatest reduction in SOD and catalase levels, as shown in Table 3.

**Table 3:** Effect of lenvatinib, ZnO-NPs and cotherapy of ZnO-NPs + lenvatinib on oxidative stress and inflammatory marker

Groups	ROS (pg/mg)	MDA (nmol/mg)	Catalase (ng/mg)	SOD (pg/mg)	TNF-α (pg/mg)	*P* value
SEC	123 ± 1.8	0.9 ± 0.17	8.7 ± 0.56	171.7 ± 2.1	102 ± 2.1	
Lenvatinib	217 ^a^ ± 10.9	3.2 ^a^ ± 0.7	4.8 ^a^ ± 0.97	100 ^a^ ± 14.6	220 ^a^ ± 10.4	*P* = 0.001
ZnO-NPs	302 ^ab^ ± 3.6	5.2 ^ab^ ± 0.27	3.1 ^ab^ ± 0.18	53.4 ^ab^ ± 2.2	350 ^ab^ ± 4.5	*P* = 0.001
Lenvatinib + ZnO-NPs	374^abc^ ± 3.04	6.2^abc^ ± 0.17	1.4^abc^ ± 0.18	38.4^abc^ ± 1.6	422 ^abc^ ± 1.9	*P* = 0.001

Data is represented as mean ± SD (n = 6). (n = 6). denotes probability, ^a^ Significant vs SEC, ^b^ Significant vs lenvatinib, ^c^ Significant vs ZnO-NPs where *P* < 0.05

### Detection of caspase-3 and caspase-9 in blood by flow cytometry

**Fig. 4:**
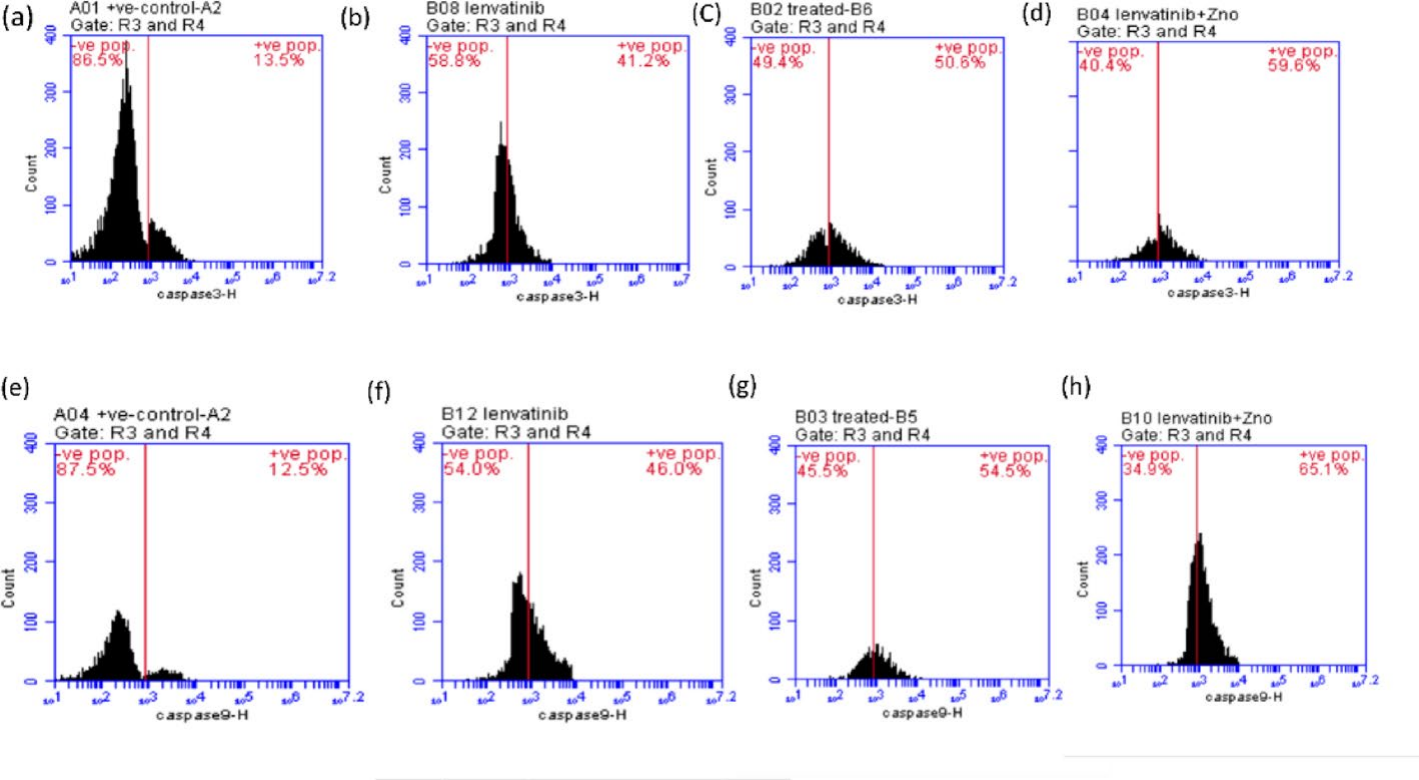
Alterations on caspase-3 and caspase-9 activities after treatment with Lenvatinib, ZnO-NPs and combined Lenvatinib and ZnO-NPs compared to untreated group. Representative flow cytometry plots of caspase-3 activity: (a) SEC group. (b) Lenvatinib group. (c) ZnO-NPs group. (d) Lenvatinib + ZnO-NPs group. Representative flow cytometry plots of caspase-9 activity: (e) SEC group. (f) Lenvatinib group. (g) ZnO-NPs group. (h) Lenvatinib + ZnO-NP group

To examine the induction of apoptosis following treatment with Lenvatinib, ZnO-NPs, and the combination of Lenvatinib and ZnO-NPs, we assessed caspase-3 and caspase-9 levels in the blood. Our results showed significant (*p* = 0.001) rise in caspase-3 (49.8 ± 2.52) and caspase-9 (54.1 ± 3.85) in Lenvatinib group comparing to SEC group. In ZnO-NPs treated group, there was a notable (*P* = 0.001) induction in levels of caspase-3 (50.46 ± 3.27) and caspase-9 (57.31 ± 1.77) compared to SEC group. However, the combination group (ZnO NPs + Lenvatinib) exhibited the higher considerable (*P* = 0.001) elevation in caspase-3 (56.5 ± 2.81) and caspase-9 (61.6 ± 2.66) in comparison to Ehrlich solid tumor bearing mice and Lenvatinib treated group as shown in Table 4 and Fig. 4.

**Table 4:** Effect of lenvatinib, ZnO-NPs and cotherapy of ZnO-NPs + Lenvatinib on the activity of caspase-3 and 9

Groups	Caspase-3	Caspase-9	*P v*alue
SEC	16.06 ± 0.53	13.1 ± 0.47	
Lenvatinib	49.8^a^ ± 2.52	54.1^a^ ± 3.85	*P* = 0.001
ZnO-NPs	50.46^a^ ± 3.27	57.31^a^ ± 1.77	*P* = 0.001
Lenvatinib + ZnO-NPs	56.5^ab^ ± 2.81	61.6^ab^ ± 2.66	*P* = 0.001

Data is represented as mean ± SD (n = 6)

*P* denotes probability, ^a^ Significant vs SEC, ^b^ Significant vs lenvatinib, ^c^ Significant vs ZnO-NPs where* P* < 0.05

### Detection of cell cycle analysis and active caspase-3 in tissue samples by flow cytometry

**Fig. 5:**
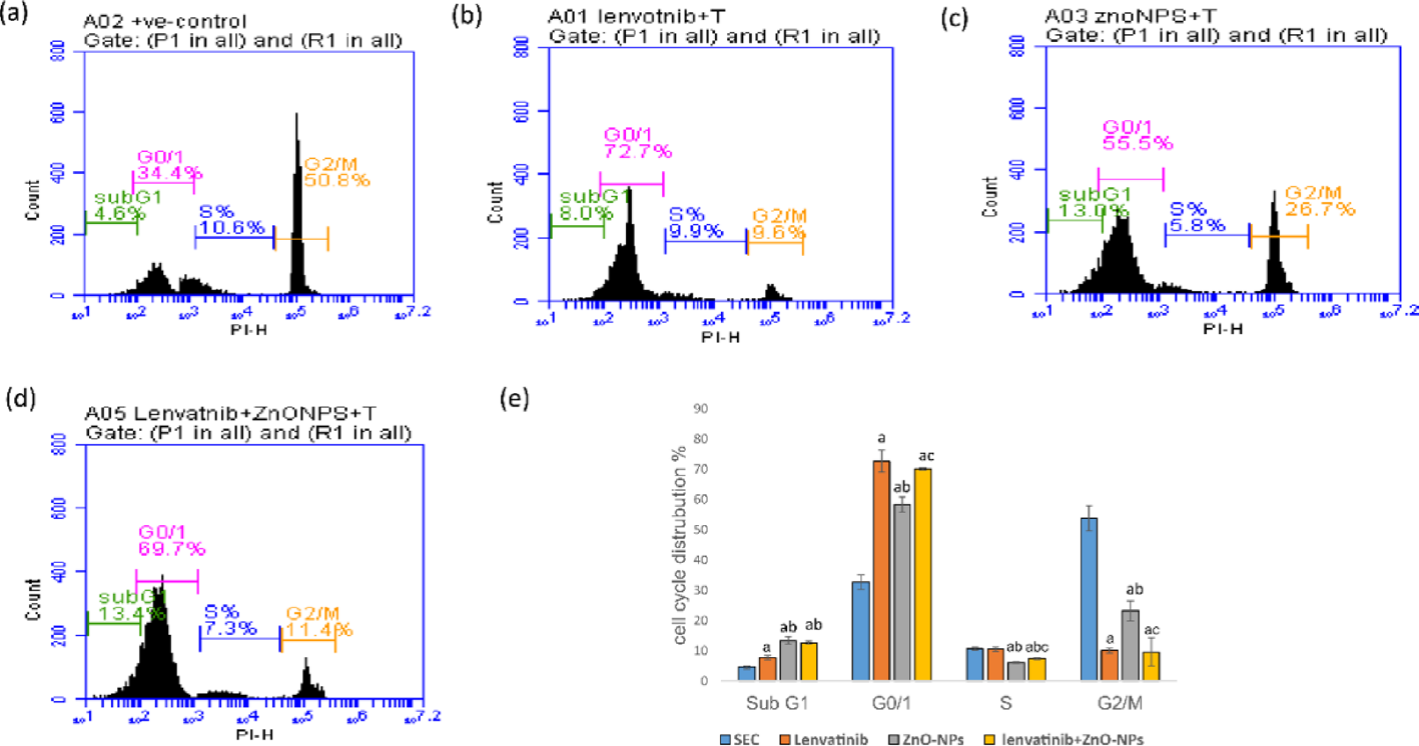
Analysis of cell cycling via flow cytometry. (a) untreated group (SEC), (b)Lenvatinib treated group, (c): ZnO-NPs treated group, (d) Lenvatinib group + ZnO-NPs. (e): cell cycle distribution %. Data are presented as mean ± SD (n = 6). Statistical significance was determined where *P* < 0.05. ^a^ Significant vs SEC, ^b^ Significant vs Lenvatinib, ^c^ Significant vs ZnO-NPs

**Fig. 6:**
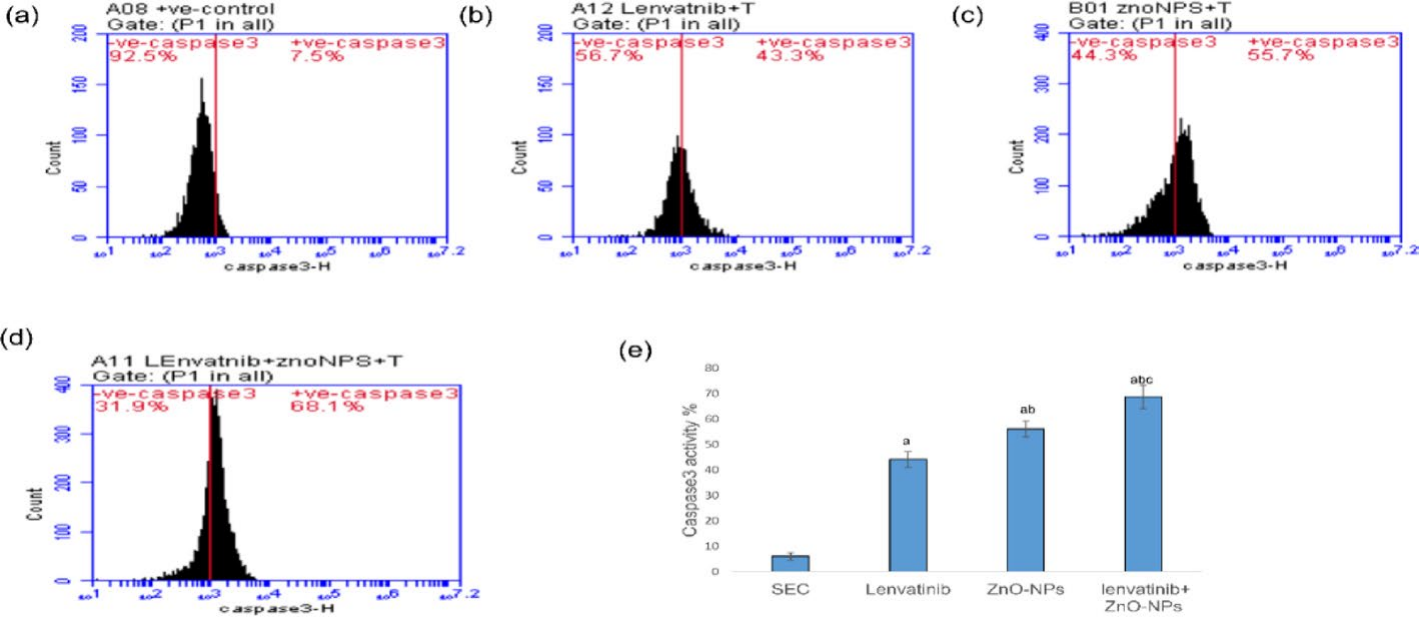
Estimation of caspase-3 activity by flow cytometry histogram, which displayed the percentage of apoptotic cells in tumor tissue across all groups. (a) SEC group. (b) Lenvatinib group. (c) ZnO-NPs group. (d) Lenvatinib + ZnO-NPs group. (e) percentage of caspase-3 activity in all treated groups. Data are presented as mean ± SD (n = 6). Statistical significance was determined where *P* < 0.05. ^a^ Significant vs SEC, ^b^ Significant vs Lenvatinib, ^c^ Significant vs ZnO-NPs

Most anticancer drugs work by inducing apoptosis in cancer cells. A cell cycle experiment in tumor tissue was carried out using flow cytometry to determine whether apoptosis was induced in the treatment groups. In Lenvatinib-treated group, there was a substantial rise (*P* = 0.0001) in the SubG1 and G0/1 populations (7.7% and 72.7, respectively), and a significant drop (*P* = 0.0001) in the G2/M population (10.1%), but there was no significant change (*P* = 0.9) in the S population (10.5%) compared to the SEC group. In ZnO-NPs treated groups there was a marked rise seen *(P* = 0.001) in the Sub G1and G0/1 populations (13.4% and 58.4% respectively) and a notable arrest (*P* = 0.0001) in the S and G2/M phases (6.08% and 23.2% respectively). In combination treated group (ZnO-NPs + Lenvatinib), there was a substantial rise (*P* = 0.0001) recorded in Sub G1 and G0/1 phases (12.7% and 70.2% respectively) and a more pronounced considerable arrest (*P* = 0.0001) in the S and G2/M phases (7.55% and 9.6% respectively) as shown in Fig. 5. In addition, the activity of caspase-3 recorded a significant increase (*P* = 0.0001) in all treated groups with a particularly marked elevation observed in the combination group (ZnO-NPs + Lenvatinib) (79.0%) in comparison to all treated groups Fig. 6.

### Expression of BcL2 and cyclin D1 by western blotting

**Fig. 7:**
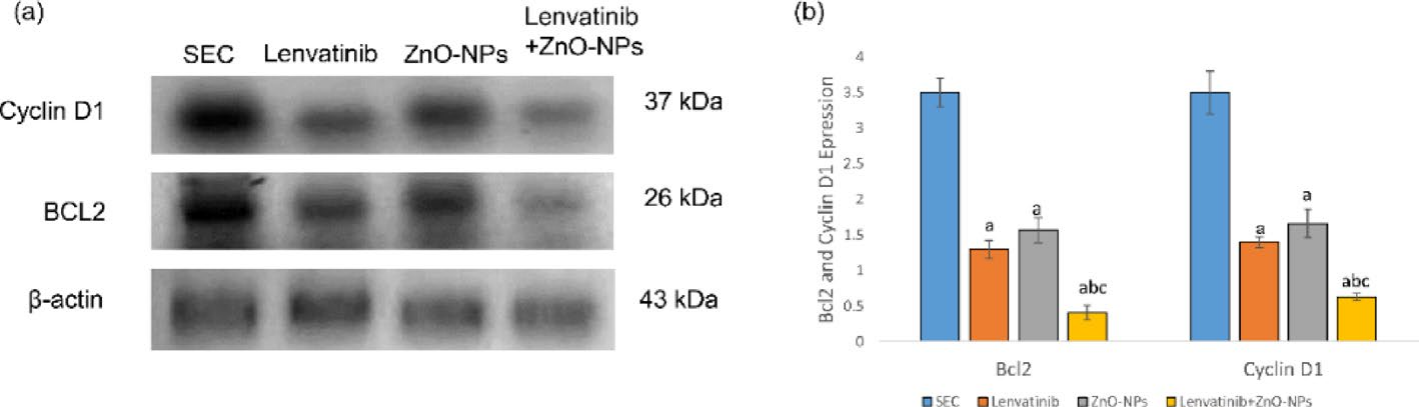
(a) Bcl-2 and Cyclin D1 protein expression in Ehrlich solid tumor tissues were examined using Western blot in untreated and treated group. An effective loading control was β-actin. (b) shows the relative protein expression of both Bcl2 and Cyclin D1in solid Erlich tumor tissues after treatment with Lenvatinib, ZnO-NPs and Lenvatinib + ZnO-NPs in comparison to SEC group. Data are expressed as mean ± SD, n = 3 biological replicates per group, ^a^ Significant vs SEC, ^b^ Significant vs Lenvatinib, ^c^ Significant vs ZnO-NPs where* P* < 0.05

Western blot analysis revealed significant alterations in BcL2 and Cyclin D1 expression in tumor tissues. As shown in Fig. 7, after oral gavage of Lenvatinib in mice bearing SEC, the expression of anti-apoptotic Bcl2 protein was remarkably reduced (1.3±0.13) while expression level of cyclin D1 protein was remarkably downregulated (1.4 ± 0.04) relative to SEC group. On the other hand, in ZnO-NPs treated group, there was a notice decrease in expression of BcL2 protein (1.57 ± 0.17) but the level of cyclin D1 protein considerably decreased (1.66 ±0.19) in comparison with SEC group. The cotherapy treated group (ZnO-NPs + Lenvatinib) exhibited the lowest expression in both Bcl2 and Cyclin D1 proteins (0.4± 0.09 and 0.63±0.05, respectively) compared to all groups. Uncropped Western blot images are included in the Supplementary File Fig. S1

### Histopathological investigations for solid tumors

**Fig. 8:**
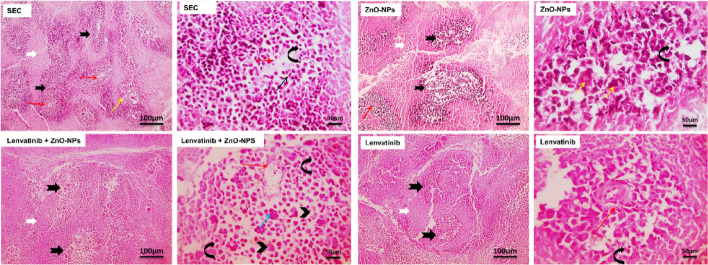
Representative images for H&E staining of tumor sections from SEC mice, ZnO-NPs, Lenvatinib, and combination between ZnO-NPS + Lenvatinib treated mice. Low magnification: 100x, bar 100; high magnification: 400x, bar 50

**Fig. 9:**
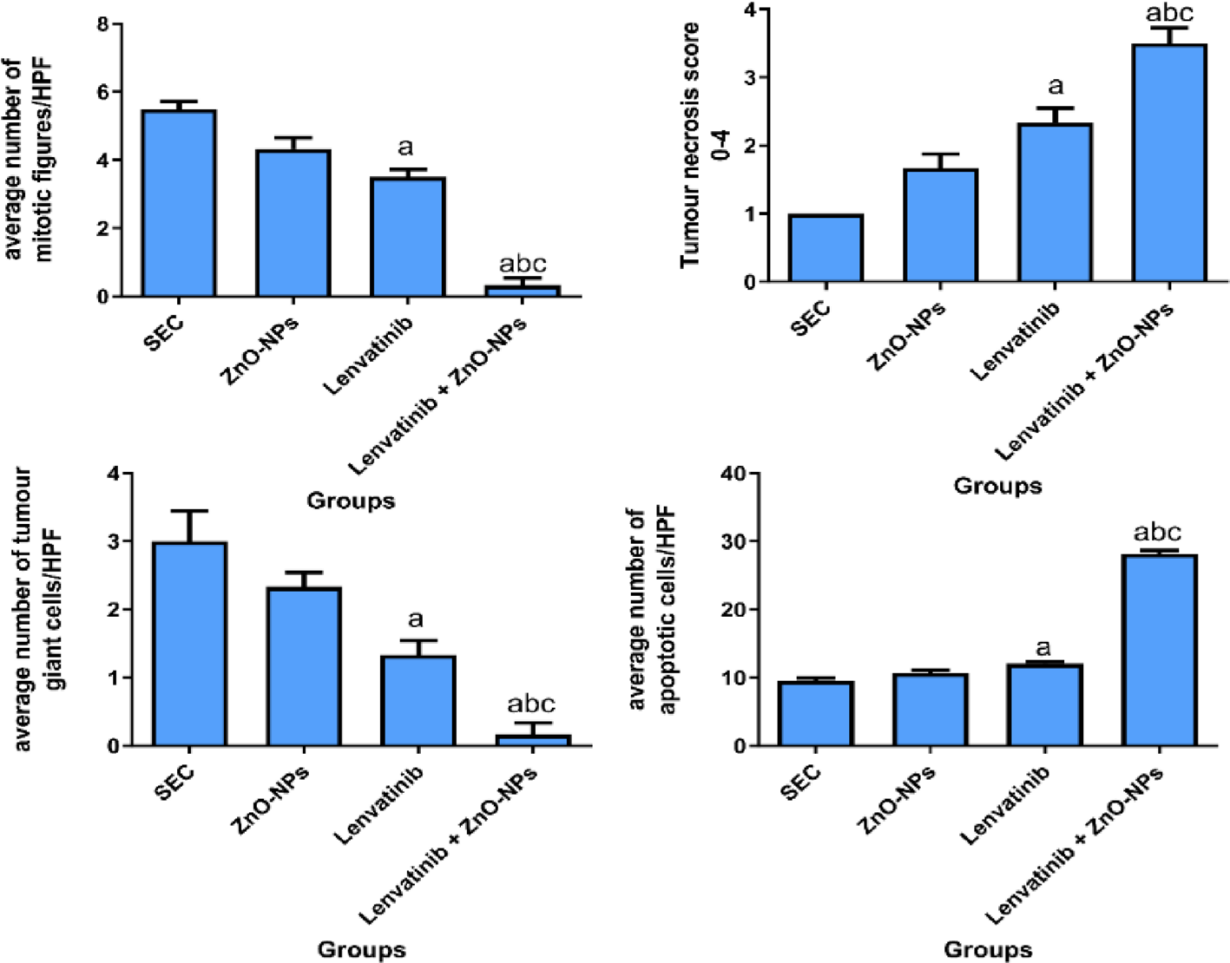
Semiquantitative scoring of tumor necrosis in different groups. The average number of mitotic figures, tumor giant cells and apoptotic tumor cells. Values represented as mean ± SD (n = 6). ^a^ Significant vs SEC, ^b^ Significant vs Lenvatinib, ^c^ Significant vs ZnO-NPs where* P* < 0.05

As shown in Fig. 8, light micrographs of the entire solid tumor sections from SEC showed high tumor growth rate as indicated by multiple large sized viable areas (thick black arrows) surrounded by small pale eosinophilic structure-less necrotic areas (thick white arrows). The viable areas consisting of pleomorphic EAC cells having aberrant nuclei (thick black arrows), atypical mitotic figures (thin black arrow) and many tumor giant cells (yellow arrows) around newly formed blood capillaries (red arrows) with few apoptotic cells (curved arrows). Solid tumor sections from **ZnO-NPs treated mice** showed fewer viable areas (thick black arrows) surrounded by wider pale eosinophilic structure-less necrotic areas (thick white arrows). The viable areas consist of pleomorphic EAC cells having aberrant nuclei (thick black arrows) and some tumor giant cells (yellow arrows) around newly formed blood capillaries (red arrows) with few apoptotic cells (curved arrows). Solid tumor sections from **Lenvatinib treated mice** showing fewer viable areas (thick black arrows) surrounded by wider pale eosinophilic structure-less necrotic areas (thick white arrows). The viable regions include pleomorphic EAC cells with atypical nuclei (thick black arrows) surrounding recently developed blood capillaries (red arrows) with few apoptotic cells (curved arrows). Solid EAC tumor sections from **Lenvatinib + ZnO-NPs treated mice** showing the lowest tumor growth rate as indicated by fewer viable areas (thick black arrows) surrounded by wider pale eosinophilic structure-less necrotic areas (thick white arrows). The viable areas consist of decreasing numbers of shrunken EAC cells (thick black arrows), many ghost cells that completely lost cellular details (black arrowheads) with few apoptotic cells (curved arrows) and foci of calcification (blue arrows) surrounding recently developed blood capillaries (red arrows). Semiquantitative scoring of tumor necrosis, number of mitotic figures, tumor giant cells and apoptotic tumor cells in different groups are indicated in Fig. 9.

### Histopathological investigations for organs

To investigate side effects of ZnO-NPs, Lenvatinib and combination between ZnO-NPS + Lenvatinib after treatment for 4 weeks, H&E staining was used for histological examination of major organs like liver, kidney, heart and spleen. In Fig. S4, light micrographs of the liver sections from normal mice showing normal hepatic architecture. Liver sections from SEC mice showing dilation of portal vessels, edema & marked extramedullary hematopoiesis in portal areas, diffuse micro and macro vesicular steatosis in many hepatocytes. Liver section from different treatment groups showing: dilation of portal vessels and sinusoids, decreased extramedullary hematopoiesis in portal areas, mild micro-vesicular steatosis in few hepatocytes in ZnO-NPs treated mice, dilation of portal vessels, decreased extramedullary hematopoiesis in portal areas, diffuse micro-vesicular steatosis in many hepatocytes in Lenvatinib treated mice, normal hepatocytes, normal portal vessels, mild focus of extramedullary hematopoiesis in sinusoids in ZnO-NPs + Lenvatinib treated mice. In Fig. S5, Light micrographs of the kidney section from normal mice showing normal glomeruli, tubules and interstitial tissue. Kidney section from SEC mice showing tubular dilation & atrophy, interstitial edema & inflammation, distorded glomeruli characterized by shrinkage & atrophy of glomerular tuft and dilation of Bowman's capsule with proteaceous material. Kidney section from different treatment groups showing similar lesions as in SEC mice with less extent in ZnO-NPs treated mice, normal tubules and glomeruli with mild vascular congestion, perivascular edema and inflammation in Lenvatinib treated mice, normal appearance of tubules and glomeruli with very mild interstitial edema and inflammation in ZnO-NPs + Lenvatinib treated mice. In Fig. S6, Light micrographs of the heart section from normal mice showing normal muscle fibers with minimal interstitial space. Heart section from SEC mice showing marked shrinkage and atrophy muscle fibers with markedly increased interstitial space besides leukocytic cells infiltration. Heart section from different treatment groups showing marked shrinkage and atrophy muscle fibers with decreased interstitial space in ZnO-NPs treated mice, normal muscle fibers with markedly decreased interstitial space in Lenvatinib treated mice, normal muscle fibers and minimal interstitial space in ZnO-NPs + Lenvatinib treated mice. In Fig. S7, Light micrographs of the spleen section from normal mice showing normal splenic architecture with well-defined lymphoid follicles and normal red pulp. Spleen section from SEC mice showing ill-defined splenic parenchyma with diffuse white pulp, distorted lymphoid architecture due to severe depletion of lymphocytes from both white &red pulps. Spleen section from different treatment groups showing similar lesions as in SEC mice with less extent in ZnO-NPs treated mice, normal splenic architecture with well-defined lymphoid follicles with increased lymphocytic cells population in both white &red pulps in Lenvatinib treated mice and ZnO-NPs + Lenvatinib treated mice.

### Immunohistochemical assessment

**Fig. 10:**
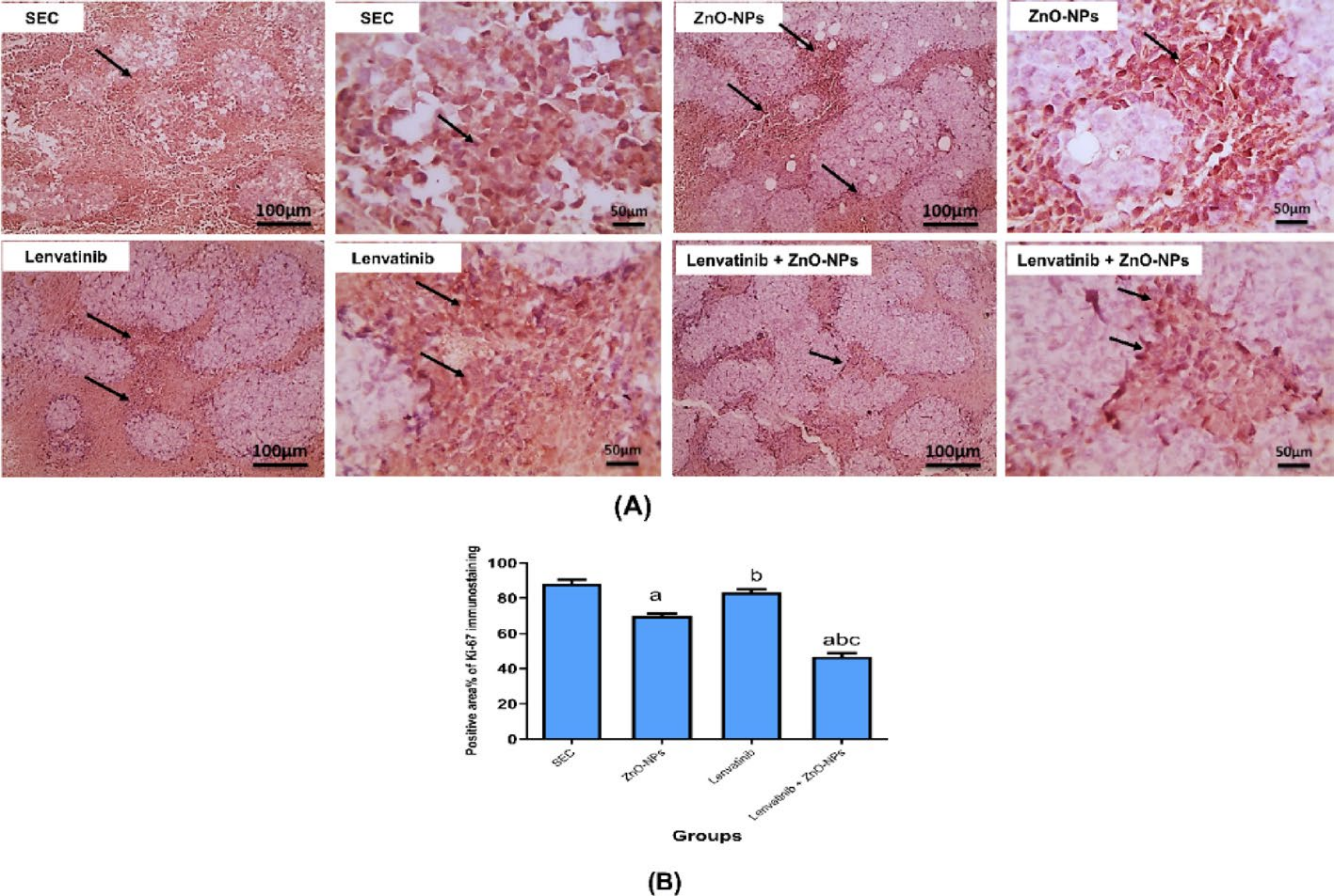
(A) Effect of Lenvatinib, ZnO-NPs and ZnO-NPs + Lenvatinib on Ki-67 positive immunostaining in tumor sections. X:100 bar 100 and X:400 bar 50. (B) Bars are values of mean ± SD (n = 6) for area% of Ki-67 positive immunostaining in tumor sections. ^a^ Significant vs SEC, ^b^ Significant vs Lenvatinib, ^c^ Significant vs ZnO-NPs where *P* < 0.05

As shown in Fig. 10A, Microscopic pictures of immunostained tumor sections against Ki-67 from positive control group showed wide positive brown area of immunostaining. Tumor sections from Lenvatinib group showed slightly decreased positive brown area of immunostaining. Tumor sections from ZnO-NPs treated group showed moderately decreased positive brown area of immunostaining. Tumor sections from ZnO-NPs + Lenvatinib group showed the smallest positive brown area of immunostaining. In Fig. 10B, significant decreases in Ki-67 positive immunostaining were seen in ZnO-NPs group and combination group, with the lowest level detected in the ZnO-NPs + lenvatinib group compared with the SEC group. Black arrows showed Ki-67 positive tumor cells (Fig. 10A).

## Discussion

Cancer is characterized by the uncontrolled proliferation of abnormal cells that can invade surrounding tissues and metastasize to distant organs. Conventional therapeutic strategies, including chemotherapy and radiotherapy, remain the cornerstone of cancer treatment; however, their clinical application is often limited by severe adverse effects and systemic toxicity. Therefore, the development of safer and more effective anticancer therapies remains a major challenge in oncology research [41]. Recently, nanotechnology-based approaches have shown considerable promise in cancer diagnosis and treatment. Among various nanomaterials, ZnO-NPs have attracted significant attention because of their selective cytotoxicity toward cancer cells, biocompatibility, ease of synthesis, and potent anticancer activity [42]. Lenvatinib, an orally administered multi target tyrosine kinase inhibitor (TKI), was approved by the FDA in 2018 as a first line treatment for advanced hepatocellular carcinoma (HCC). Lenvatinib exerts its therapeutic activity through inhibition of several receptor tyrosine kinases, including FGFR1–4, VEGFR1–3, PDGFRα, KIT, and RET [43]. Nevertheless, the development of resistance to lenvatinib has been frequently reported in cancer patients [44]. This resistance may arise through activation of alternative survival pathways such as PI3K/AKT and MAPK/ERK signaling, in addition to enhanced antioxidant defense mechanisms [45]. Consequently, combination therapy may represent an effective strategy to enhance the antitumor efficacy of lenvatinib. Therefore, the present study investigated the antitumor effects of ZnO-NPs and lenvatinib, individually and in combination, using the Ehrlich solid carcinoma (ESC) model, which closely resembles human cancers in terms of rapid growth, high transplantability, and immune evasion [46].

Physicochemical characterization confirmed the successful synthesis of ZnO-NPs with nanoscale dimensions, excellent colloidal stability, and a crystalline wurtzite structure. These physicochemical properties are critical because nanoparticle size, crystallinity, surface charge, and surface chemistry greatly influence cellular uptake, dispersion, reactive oxygen species (ROS) generation, and ultimately the anticancer efficacy of ZnO-NPs.

Our findings demonstrated a marked reduction in tumor volume and a significant increase in tumor growth inhibition percentage in the Lenvatinib treated and combination treated groups (ZnO-NPs + lenvatinib) compared with the SEC group. These effects may be attributed to the antiangiogenic activity of lenvatinib through inhibition of VEGFR/FGFR dependent signaling pathways [47], as well as suppression of FGFR-FRS2-MAPK mediated survival signaling. In parallel, ZnO-NPs induce ROS mediated mitochondrial dysfunction, leading to activation of the p53/Bax pathway and caspase dependent apoptosis in tumor cells [48]. These findings are consistent with Wang et al. (2018), who reported that the combination of lenvatinib and I-131 significantly suppressed tumor growth in nasopharyngeal cancer xenografts [49]. Similarly, Shorbagy et al. (2019) demonstrated a significant reduction in tumor size in mice bearing Ehrlich solid tumors following ZnO-NPs treatment [50].

Hematological analysis revealed no major abnormalities except for a significant reduction in hemoglobin levels in SEC bearing mice, which is consistent with cancer associated anemia [51]. Interestingly, hemoglobin levels significantly improved in the combination-treated group. This improvement may be attributed to the synergistic antitumor effects of lenvatinib and ZnO-NPs, which reduced tumor burden and alleviated malignancy-associated systemic stress. These findings are in agreement with Nabil et al., who reported a significant increase in hemoglobin levels following treatment of solid tumor-bearing mice with sorafenib and ZnO-NPs, either alone or in combination [24].

The progression of Ehrlich solid tumors is frequently associated with metabolic disturbances and organ dysfunction, resulting in elevated serum levels of AST, ALT, urea, and creatinine [52]. ALT is considered more specific for hepatocellular injury, whereas AST may also increase due to muscle damage and tissue destruction associated with tumor proliferation [53]. This explains the significant elevation of ALT and AST observed in the SEC group compared with the normal control group. Treatment with lenvatinib, ZnO-NPs, or their combination significantly improved liver enzyme levels. Moreover, serum creatinine levels were significantly modulated in all treated groups, while urea levels showed marked improvement particularly in the combination group. These findings suggest that the treatments, especially the combined regimen, may alleviate tumor-associated hepatic and renal dysfunction through effective suppression of tumor progression and reduction of malignancy-induced systemic stress. These observations are generally consistent with Radwan et al. (2021), who reported that ZnO-NPs nanocomposites significantly attenuated doxorubicin-induced toxicity and improved liver and kidney functions in tumor bearing animals [54].

Reactive oxygen species play a dual role in cancer biology; however, excessive ROS generation can induce cancer cell death through oxidative stress and suppression of antioxidant defense systems [55]. In the present study, all treated groups exhibited increased ROS and MDA levels accompanied by depletion of antioxidant enzymes, including SOD and CAT, compared with the untreated SEC group. Additionally, TNF-α levels were significantly elevated following treatment with lenvatinib, ZnO-NPs, and their combination. ZnO-NPs may induce ROS generation through activation of intracellular redox reactions and stimulation of inflammatory responses toward nanoparticles, leading to depletion of antioxidant defenses and induction of inflammatory mediators [56]. Furthermore, lenvatinib may contribute to ROS accumulation through inhibition of FGFR4 signaling. These findings are supported by Motafeghi et al., who demonstrated that ZnO-NPs induced ROS generation, reduced intracellular glutathione, and elevated MDA levels in prostate and colon cancer cell lines [57], Likewise, Wang et al. reported that combined treatment with vincristine and lenvatinib resulted in excessive intracellular ROS accumulation in liver cancer cells, associated with activation of TNF-α-related inflammatory and apoptotic pathways [58].

Apoptosis is tightly regulated by caspases and Bcl-2 family proteins and is essential for maintaining tissue homeostasis. While Bcl-2 inhibits intrinsic apoptosis, caspase-3 and caspase-9 play central roles in the execution of apoptotic signaling [59, 60]. In the current study, treatment with lenvatinib, ZnO-NPs, and particularly their combination significantly elevated caspase-3 and caspase-9 levels in blood and increased caspase-3 expression in tumor tissue. These findings indicate strong activation of intrinsic mitochondria-mediated apoptosis. ZnO-NPs are known to induce ROS-mediated mitochondrial damage, resulting in cytochrome c release and activation of apoptotic protease activating factor-1 (Apaf-1), caspase-9, and caspase-3 [56]. Meanwhile, lenvatinib promotes apoptosis through activation of caspase-dependent pathways and inhibition of ERK and PI3K/AKT survival signaling [61]. These results are consistent with Enomoto et al., who reported increased cleaved caspase-3 expression in anaplastic thyroid cancer models treated with lenvatinib [62]. Similarly, Akhtar et al. (2012) demonstrated elevated caspase-3 expression in HepG2 cells following ZnO-NPs exposure [63].

Cell cycle analysis further supported these findings. Treated groups, especially the combination group, demonstrated marked accumulation of cells in the G0/G1 phase accompanied by reduction in the G2/M phase, indicating cell cycle arrest and inhibition of cellular proliferation. These results are consistent Radwan et al. (2021), who reported G0/G1 cell cycle arrest following ZnO-NPs treatment in Ehrlich ascites carcinoma models, and Lee et al. (2018), who demonstrated lenvatinib-induced G0/G1 arrest in tumor xenograft models [54, 64].

Moreover, Bcl-2 expression was markedly upregulated in the SEC group but significantly downregulated following treatment with ZnO-NPs, lenvatinib, and their combination. This reduction, together with the observed increase in caspase-3 and caspase-9 levels, suggests a shift in the Bcl-2 family balance toward pro-apoptotic mitochondrial signaling. These findings are in agreement with Tong et al. (2024), who reported downregulation of Bcl-2 expression in gastric cancer following lenvatinib treatment [65].

Cyclin D1, a critical regulator of G1/S phase transition, was highly expressed in SEC tumor tissue but significantly reduced in the treated groups, particularly in the combination group. The suppression of cyclin D1 further confirms induction of G0/G1 cell cycle arrest and inhibition of tumor proliferation. These findings are supported by Alipour et al. (2022), who demonstrated significant downregulation of cyclin D1 expression in ovarian cancer cells following ZnO-NPs treatment [66]. Similarly, another study reported reduced cyclin D1 expression in hepatocellular carcinoma cells after lenvatinib administration [67].

Ki-67, a well-established marker of cellular proliferation, was significantly reduced in the combination-treated group, further confirming effective suppression of tumor growth. These results are in line with previous studies by Gu and Yang (2024) and Revilla et al. (2023), who observed decreased Ki-67 expression following treatment with ZnO-NPs and lenvatinib, respectively [68, 69].

The molecular and biochemical findings were further validated by histopathological examination. Tumor tissues from the SEC group exhibited marked cellular atypia, intense mitotic activity, and aggressive proliferation. In contrast, tumor sections from the combination-treated group demonstrated extensive necrosis, apoptotic bodies, and markedly reduced viable tumor cells, indicating potent antitumor activity. These findings are consistent with Aly et al. (2025), who observed focal necrotic areas in tumor tissues following ZnO-NPs treatment [70].

**Fig. 11:**
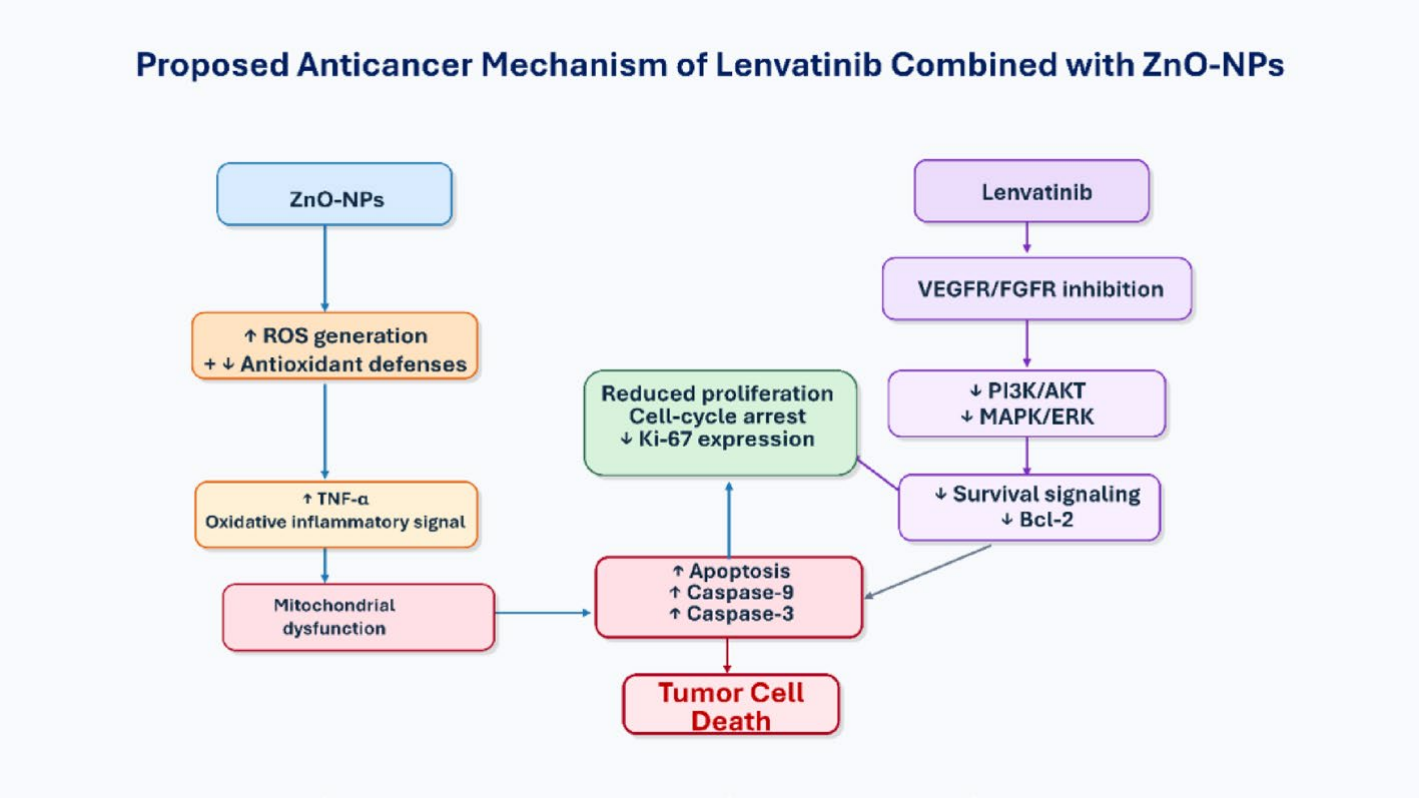
Proposed Anticancer Mechanism of Lenvatinib Combined with ZnO-NPs

Although ZnO-NPs possess promising anticancer properties, excessive ROS generation may potentially induce toxicity in normal tissues. Nevertheless, histopathological examination of the liver, kidney, heart, and spleen demonstrated preservation of normal tissue architecture in the treated groups, particularly in the combination group, suggesting favorable biocompatibility and minimal systemic toxicity. Overall, our findings suggest that, as illustrated in Fig. 11, the combination of lenvatinib andZnO nanoparticles exerts synergistic antitumor activity by simultaneously targeting oxidative stress and oncogenic survival pathways. ZnO nanoparticlestrigger ROS-mediated mitochondrial apoptosis, whereas lenvatinib suppresses VEGFR/FGFR-dependent PI3K/AKT and MAPK/ERK signaling, leading toreduced proliferation, decreased Ki-67 expression, enhanced caspase activation, and increased tumor cell apoptosis. Collectively, these complementarymechanisms highlight the potential of this combination therapy as a promising anti-cancer strategy and warrant further preclinical and clinicalinvestigation.

## Conclusion

In Ehrlich solid tumor-bearing mice, the combination of ZnO-NPs and Lenvatinib showed significant anticancer activity by inducing apoptosis, reducing proliferation, lowering angiogenesis, and enhancing liver function, while systemic toxicity was ameliorated. These findings support additional research into this combination therapy as a potential clinical strategy for cancer treatment.

## Supplementary Information

Below is the link to the electronic supplementary material.

Supplementary Material 1

## Data Availability

All data generated or analysed during this study are included in this published article and its supplementary information.
