# Supplementary material for: Combined effects of Lenvatinib and zinc oxide nanoparticles by promoting apoptosis and anti-proliferative activity in mice bearing Ehrlich solid tumors
Source: Discov Nano. 2026 Jul 20;21(1):354. doi: 10.1186/s11671-026-04802-4 (PMC13381423; doi:10.1186/s11671-026-04802-4)
Supplement: Supplementary file 1 — Supplementary Material 1 [file 11671_2026_4802_MOESM1_ESM.docx]

**Fig S 1:** Uncropped Western blot images in section (1), (2) and (3) **A**: The first original blot PVDF membrane showed the target protein BCL2 which is normalized versus housekeeping protein beta actin. **B**: The first original blot PVDF membrane showed the target protein Cyclin D1 which is normalized versus housekeeping protein beta actin**. C**: The first original blot PVDF membrane showed the house keeping protein beta actin.


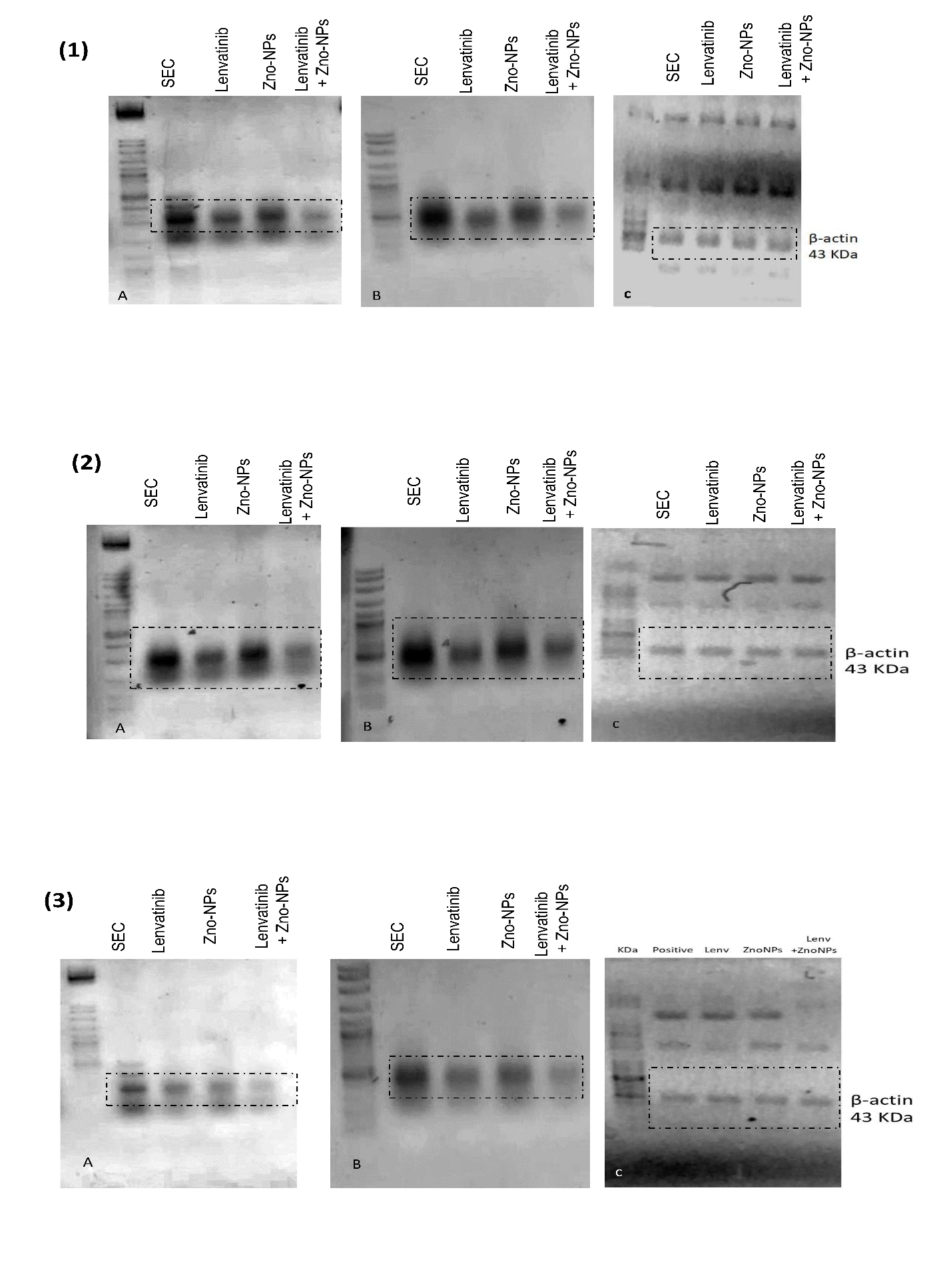


**Fig S 2:** Caspase-3 dot plot for Lenvatinib, ZnO-NPs and cotherapy of ZnO-NPs + Lenvatinib and unstained cell.

**
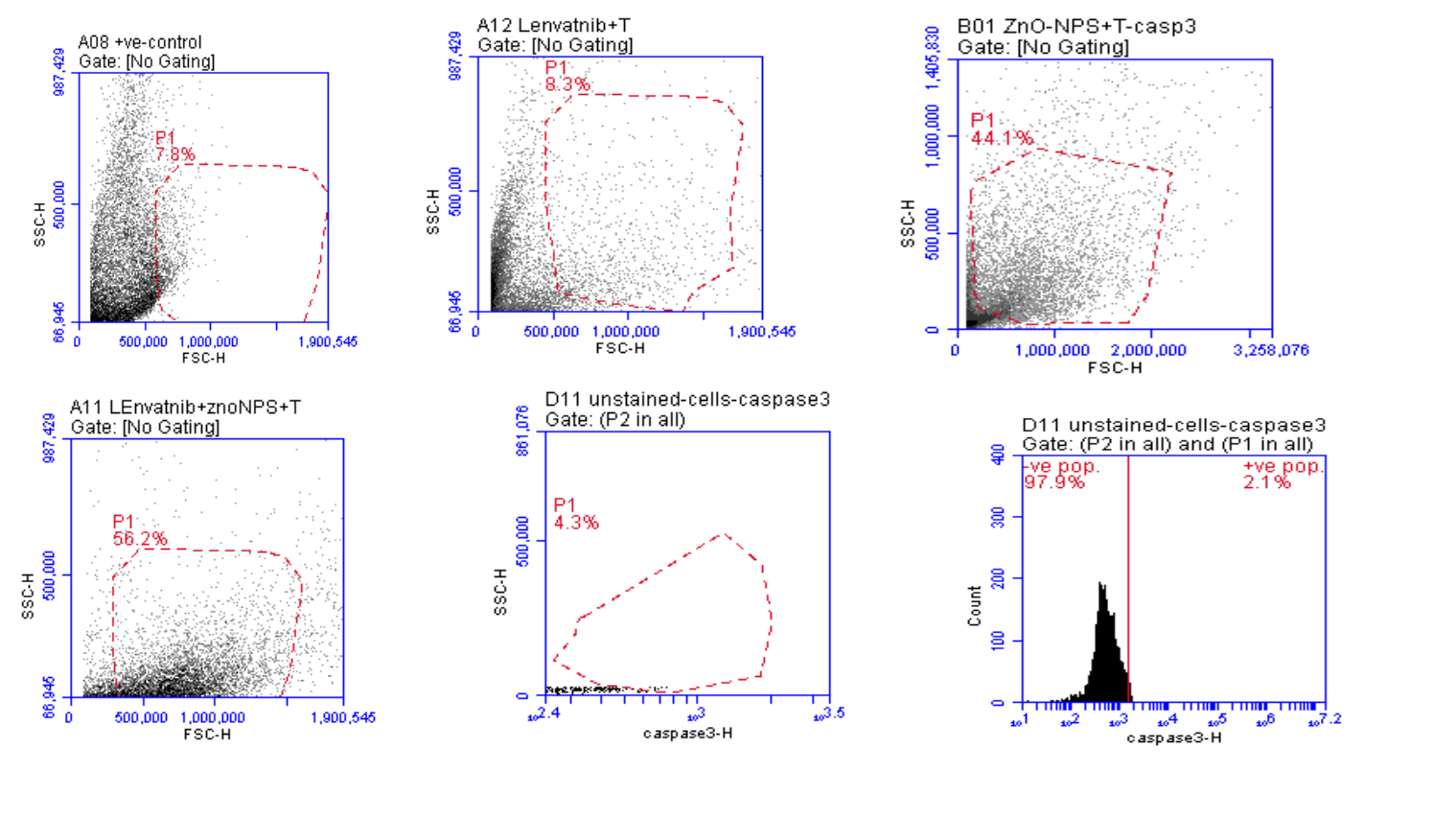
**

**Fig S 3: Dot plot of cell cycle analysis** Lenvatinib, ZnO-NPs and cotherapy of ZnO-NPs + Lenvatinib

**
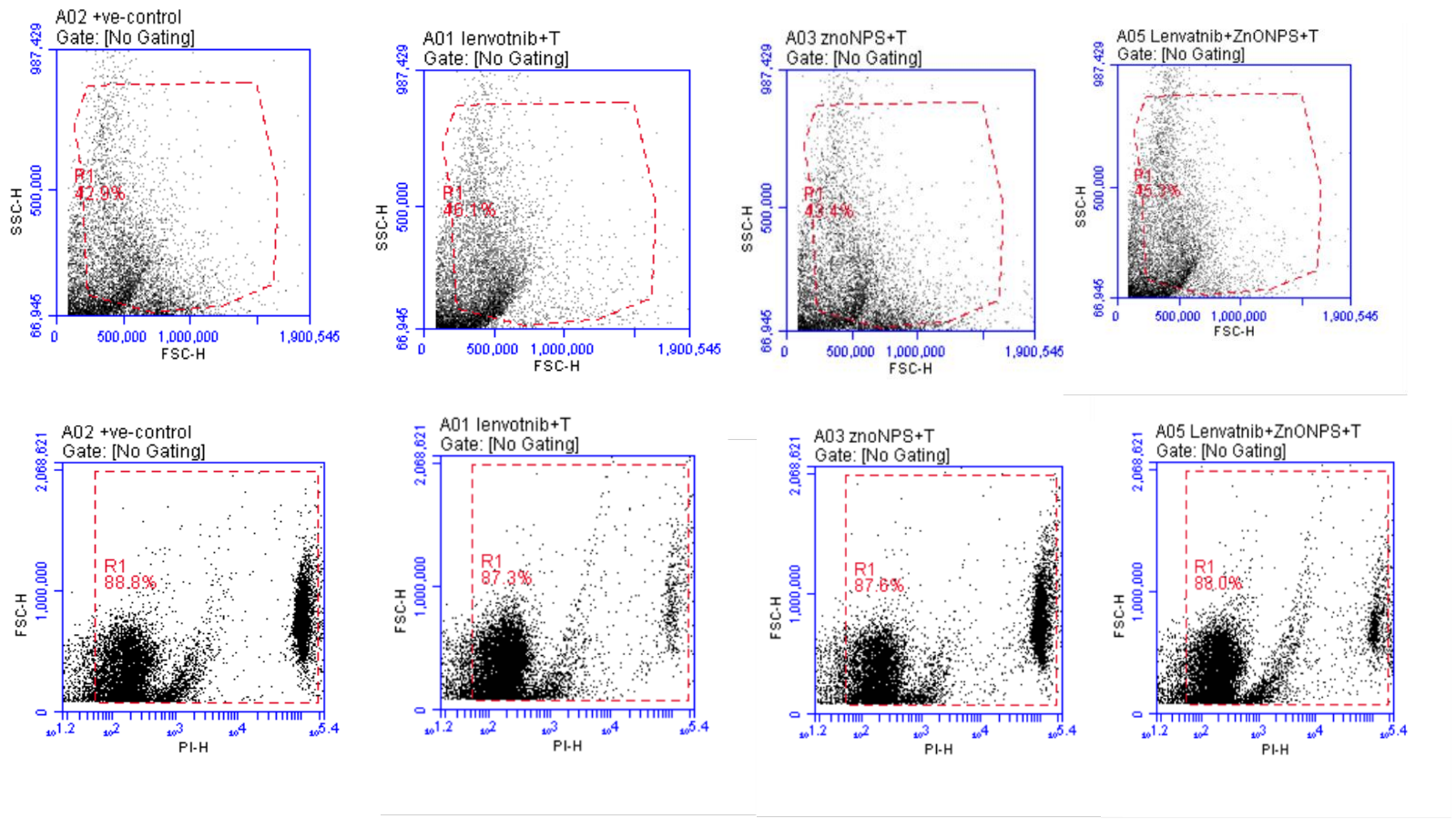
**

**Fig S 4**: Normal mice showed preserved liver architecture. SEC mice showed portal vessel dilation, edema, marked extramedullary hematopoiesis, and fatty changes. ZnO-NPs- and Lenvatinib-treated mice showed variable portal dilation, reduced extramedullary hematopoiesis, and mild to diffuse steatosis. The ZnO-NPs + Lenvatinib group showed nearly normal liver architecture with minimal histopathological changes. Dilation: red arrows; edema: blue arrow; extramedullary hematopoiesis: thick black arrows; vesicular steatosis: thin black arrows. H&E Low magnification: 100x, bar 100; high magnification: 400x, bar:50.


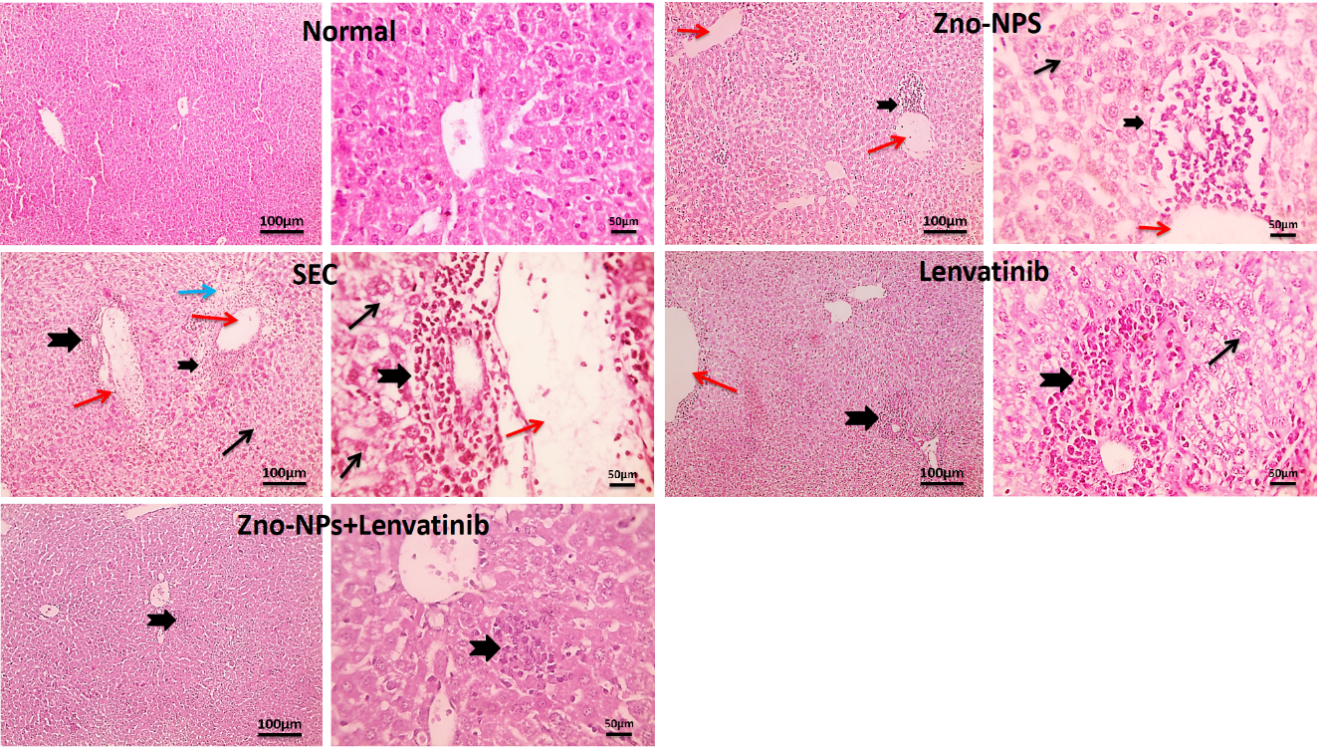


**Fig S 5:** Normal mice showed normal glomeruli, tubules, and interstitial tissue. SEC mice showed tubular dilation and atrophy, interstitial edema and inflammation, and glomerular distortion. ZnO-NPs-treated mice showed similar but milder renal changes. Lenvatinib-treated mice showed mostly normal tubules and glomeruli with mild vascular congestion, edema, and inflammation. The ZnO-NPs + Lenvatinib group showed nearly normal renal architecture with minimal interstitial edema and inflammation. Tubular dilation and atrophy: thin black arrows; interstitial edema: blue arrow; inflammation: thick black arrows; distorted glomeruli: arrowheads; vascular congestion: red arrow. H&E Low magnification: 100x, bar 100; high magnification: 400x, bar 50


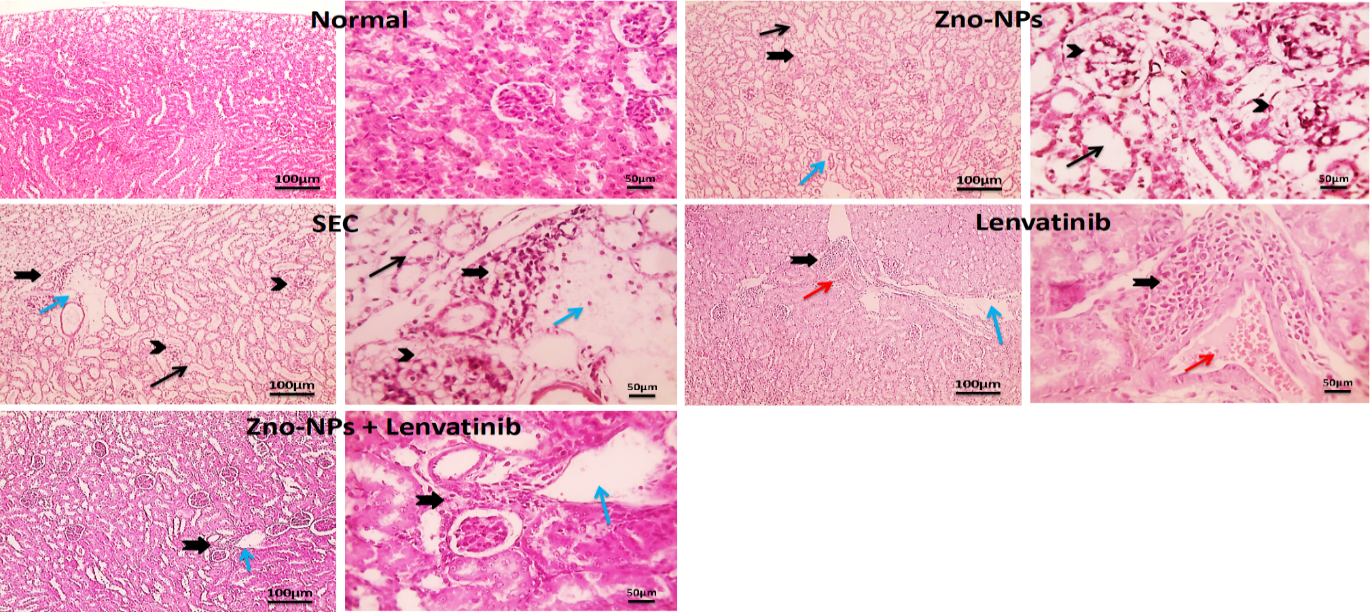


**Fig S 6**: Normal mice showed normal cardiac muscle fibers with minimal interstitial space. SEC mice showed marked muscle fiber shrinkage, increased interstitial space, and leukocytic infiltration. ZnO-NPs-treated mice showed similar but milder changes, while Lenvatinib-treated mice showed nearly normal muscle fibers with reduced interstitial space. The ZnO-NPs + Lenvatinib group showed normal cardiac architecture. Muscle fiber shrinkage and atrophy: thin black arrows; interstitial space: blue arrow; leukocytic infiltration: arrowheads. H&E, Low magnification: 100x, bar 100; high magnification: 400x, bar 50.


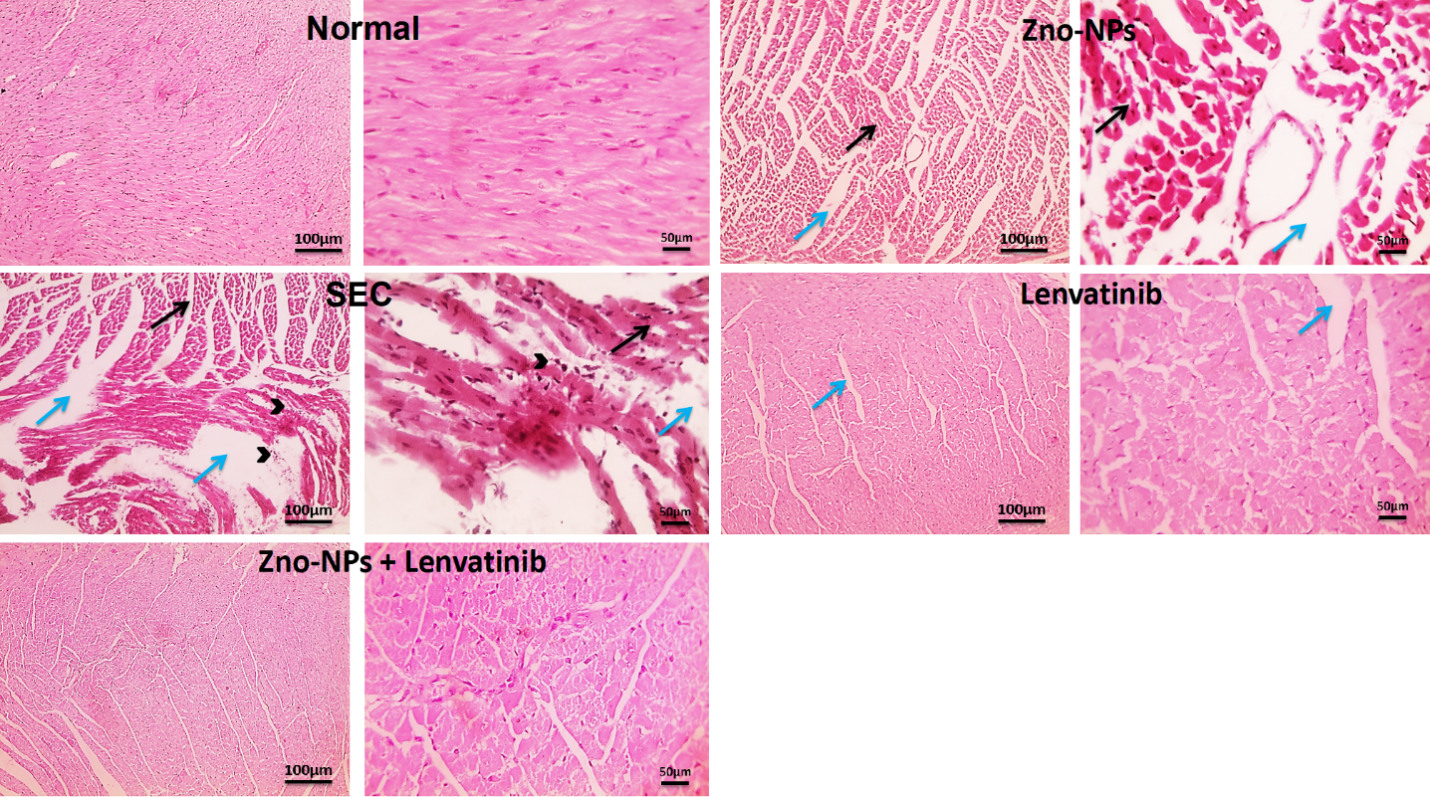


**Fig S 7:** Normal mice showed preserved splenic architecture with clear lymphoid follicles and normal red pulp. SEC mice showed disrupted splenic architecture with marked lymphocyte depletion. ZnO-NPs-treated mice showed similar but milder changes. Lenvatinib and ZnO-NPs + Lenvatinib groups showed restored splenic architecture with increased lymphocyte population. Lymphocyte depletion: thin black arrows. Low magnification: 100x, bar 100; high magnification: 400x, bar 50.


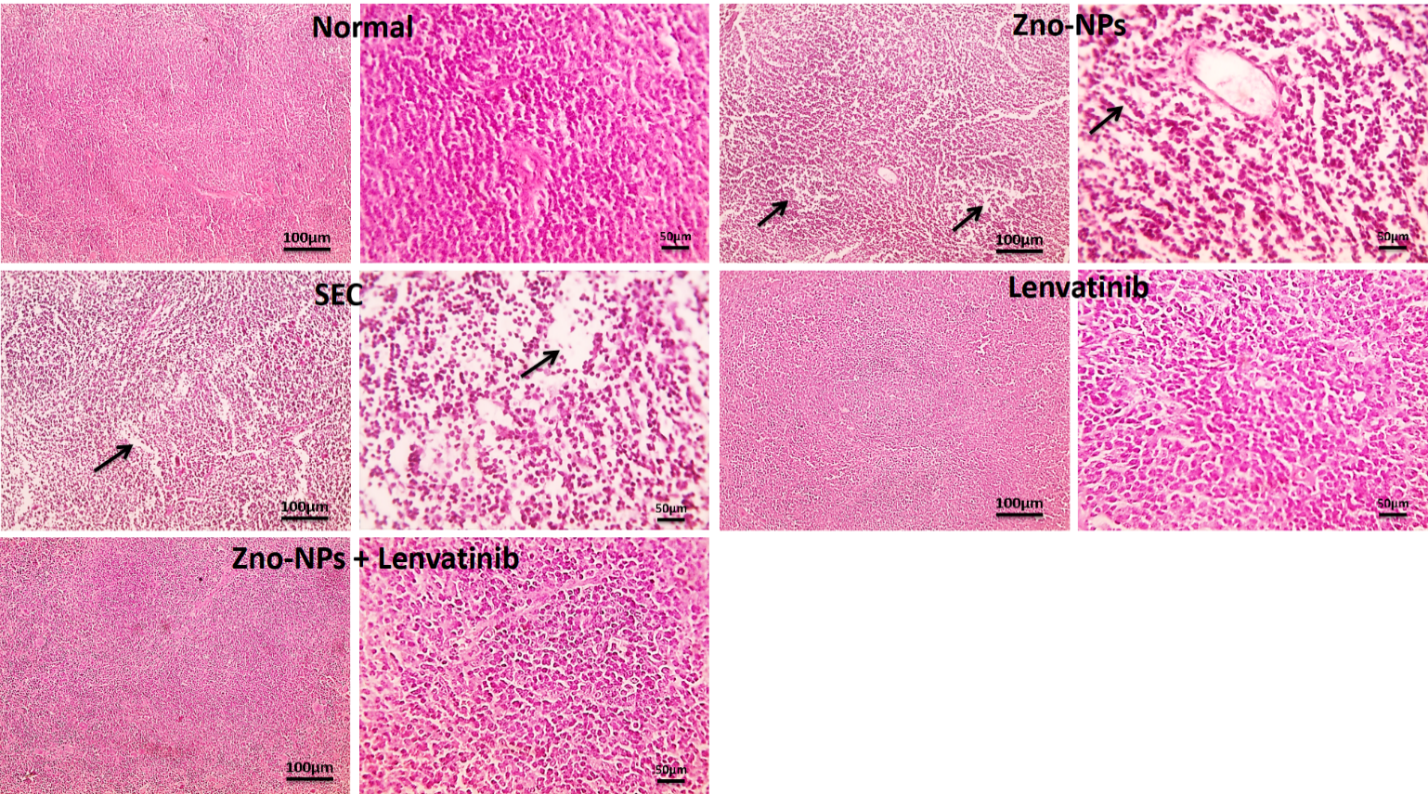


**table S 1:** FTIR band and their assignments to particular functional groups of the synthesized ZnO nanoparticles

| **Wavenumber (cm^-1^)** | **Assignment** | **Functional Group** |
| --- | --- | --- |
| 1639.68 | O-H bending vibration | Surface hydroxyl groups on ZnO surface because of adsorbed water molecules |
| 1371.56 | C–O stretching | Characteristic residual carbonate (CO_3_^-2^) species |
| 823.06 | Zn-O stretching vibration | Hexagonal ZnO crystalline structure |
| 564.20-404.51 | Zn-O stretching vibration | Metal–oxygen framework of ZnO lattice |
